# Adolescent suicide behaviors associate with accelerated reductions in cortical gray matter volume and slower decay of behavioral activation Fun-Seeking scores

**DOI:** 10.1038/s41598-025-16856-y

**Published:** 2025-09-25

**Authors:** Yi Zhou, Michael C. Neale

**Affiliations:** Virginia Institute of Psychiatric and Behavioral Genetics, Virginia Commonwealth University, Richmond, VA USA

**Keywords:** Paediatric research, Human behaviour, Motivation, Brain, Depression

## Abstract

Distinguishing those at risk of making a suicide attempt from those who experience only suicidal ideations remains a significant clinical challenge. Longitudinal studies during early adolescence may provide insight into altered brain and behavioral developmental trajectories among those who develop suicide behaviors (SB). Here, we applied linear mixed effects regression models to several global brain volumes and psychiatric/behavioral measures from the Adolescent Brain Cognitive Development (ABCD) Study. We analyzed data from baseline up until the two-year follow-up, when participants were roughly 10 to 12 years of age. Individuals who had either ever endorsed or developed SB exhibited the greatest reductions in cortical gray brain matter volume. Those who developed SB exhibited the greatest increase in DSM5-depression scores and were the only group that maintained their levels of Behavioral Activation System (BAS) Fun-Seeking behaviors. Finally, we applied a Cross-Lagged Panel Modelling approach to the whole ABCD sample and found that baseline total cortical gray matter structure significantly predicted variation in BAS Fun-Seeking behaviors at the two-year follow-up, providing evidence supportive of a potential causal relationship between these two measures. Altogether, our findings suggest that differences in total cortical gray matter volume at 9–10 years of age may impact the development of behavioral approach systems. Altered development of behavioral approach systems and depressive symptoms distinguish youth who developed suicide behaviors during early adolescence.

**Supplementary Information:**

The online version contains supplementary material available at 10.1038/s41598-025-16856-y.

## Introduction

Suicide is a leading cause of death for youths in the United States^1^. The ideation-to-action framework for suicide thoughts and behaviors (STB) encompasses many theories of suicidality which share a common premise that suicide ideation (SI) is distinct from suicide behaviors (SB), with each having its own risk factors and processes^2^. For example, Joiner’s Interpersonal Theory for Suicide (IPTS) proposes that thwarted belongingness and perceived burdensomeness contribute to suicide desire while painful and provocative events habituate an individual to the fear of pain and suicide death, thereby allowing them to acquire the capability for suicide and act on their suicide ideation^3^. Other theories further propose that developing SI requires defeat, entrapment^4^psychological pain, hopelessness, and decreased connectedness^5^while suicide capability may also include dispositional and practical risk factors such as genetic vulnerability and access to lethal means, respectively^5^. Notably, the fluid vulnerability theory (FVT) proposes that suicide risk is dynamic and depends on the interplay between acute precipitating factors and an individual’s baseline suicide risk that is determined by chronic factors^6^. Identification and validation of the factors distinguishing those at highest risk of suicide attempts is greatly needed to improve clinical diagnostic and screening tools as well establishing more effective targets of intervention.

Few studies have focused on identifying brain-based markers distinguishing those at risk of SI from SB in youth populations. Brain imaging studies in adults have reported that those with SB exhibit structural and functional alterations in brain regions broadly associated with cognitive and emotional dysregulation^7,8^. However, most of these studies are cross-sectional in nature and do not characterize factors contributing to the development of SB. Thus, there is a great need for longitudinal studies capable of characterizing changes in the brain and behaviors that may underlie the transition from SI to SB in youths.

The Adolescent Brain Cognitive Development (ABCD) study is one of the largest longitudinal studies of adolescent brain development in the world and includes almost 12,000 youths across the US and thus offers a unique opportunity to study both the development of the brain and of STB. Prior studies have already identified many risk factors associated with suicidality in the ABCD sample including discrimination stress^9^externalizing symptoms^10^sexual minority status^11^low-level alcohol use^12^altered neurocognition^13^brain structure and function^14,15^and child psychopathology and child reported family conflict^16^to name a few. The various risk factors identified in this work may reflect diverse pathways through which STB may arise in youth.

However, most of the prior studies in the ABCD study do not distinguish between those with SI only and those reporting SB, and even fewer have specifically compared brain and behavioral/psychological developmental trajectories between them. Past studies have shown that total brain volume peaks at around the end of childhood and generally decreases after this peak. This pattern is driven by decreases in cortical gray matter while white matter volumes increase throughout development^17,18^. Whether any differences in brain development coincide with the development of SB or altered trajectories in other psychiatric and behavioral measures remains to be investigated and an integration of these measures across these domains is sorely needed.

The present study aims to characterize differences in brain structure and behavioral development between youths who develop SB and those who do not. Based on the pattern of responses of participants across three timepoints in the ABCD study, we sorted individuals into a series of mutually exclusive groups. These included (i) those who developed SB and (ii) those who developed SI only. Additionally, some individuals reported STB, but their pattern of responses over time did not clearly indicate if they had developed, maintained, or even recovered from SB or SI (see Table 2 in Methods). For those individuals, we created two additional groups consisting of those who (iii) ever *endorsed SB*, or (iv) ever *endorsed SI* only. Furthermore, we included two control groups: (v) those who had never reported any STB but who exhibited clinically significant levels of depression, and (iv) a non-depressed group who also had never reported any STB. We included a depressed control group without any STB to distinguish brain and psychiatric/behavioral markers uniquely associated with STB from those associated with risk factors and processes that may be shared between depression and STB, such as psychological pain and hopelessness. Using Cross-Lagged-Panel-Modelling (CLPM), a structural equation modeling approach, we aimed to determine if there was evidence for a potential causal relationship between altered brain and psychiatric/behavioral development associated with SB.

## Results

### Sample characteristics

We summarized several demographic and clinical measures across the groups of interest from the ABCD study (Table 1). Participants were approximately 10 years of age at baseline and 12 years old at two-year follow-up. Generally speaking, participants who had ever endorsed or who developed SB exhibited higher average levels of depression and other Child-Behavior-Checklist (CBCL) scores, as well as worse socioeconomic-status (SES) conditions, than the other groups, except compared to the depressed control group which exhibited the highest CBCL scores for psychopathology and worst SES indicators in terms of poverty, combined family income, and parental education.

**Table 1:** Summary of sample characteristics.

Characteristics	*N*	Non-Depressed Controls, *N* = 9,690	Endorsed SI Only, *N* = 1,128	Developed SI Only, *N* = 488	Endorsed SB, *N* = 262	Developed SB, *N* = 158	Depressed Controls, *N* = 141	*p-value
**Sex: n (%)**	11,867							< 0.001
Male		5,039 (52)	643 (57)	208 (43)	153 (58)	65 (41)	83 (59)	
Female		4,651 (48)	485 (43)	280 (57)	109 (42)	93 (59)	58 (41)	
**Age at Baseline (weeks): Mean (SD)**	11,867	119 (8)	119 (7)	119 (7)	120 (8)	119 (7)	120 (7)	0.21
**CBCL DSM5 Depression: Mean (SD)**	11,867	1.08 (1.39)	1.96 (2.24)	2.11 (2.42)	2.99 (2.94)	3.23 (3.09)	8.74 (1.75)	< 0.001
**Percentage with clinically significant depression (CBCL DSM5 Depression scores > 98th percentile)**	11,867	0.4	5.4	6.8	11.8	13.3	100	NA
**CBCL Internalizing: Mean (SD)**	11,867	4.4 (4.1)	6.6 (5.9)	7.0 (6.3)	8.6 (6.5)	10.0 (7.9)	21.1 (6.1)	< 0.001
**CBCL Externalizing: Mean (SD)**	11,867	3.7 (4.6)	5.8 (6.1)	5.4 (5.8)	8.4 (7.7)	9.4 (8.6)	16.3 (8.6)	< 0.001
**CBCL Total Problems: Mean (SD)**	11,867	15 (14)	23 (19)	23 (19)	32 (22)	35 (26)	67 (18)	< 0.001
**Non-Suicidal Self-Injurious Behavior**,** Past or Present: n (%)**	11,801							< 0.001
Absent		9,297 (96)	913 (82)	456 (94)	156 (60)	130 (83)	134 (95)	
Present		344 (3.6)	204 (18)	28 (5.8)	105 (40)	27 (17)	7 (5.0)	
Unknown		49	11	4	1	1	0	
**Self-Reported Race/Ethnicity: n (%)**	11,865							< 0.001
White		5,093 (53)	592 (52)	236 (48)	107 (41)	82 (52)	67 (48)	
Black		1,430 (15)	169 (15)	81 (17)	63 (24)	21 (13)	17 (12)	
Hispanic		1,963 (20)	212 (19)	105 (22)	58 (22)	28 (18)	42 (30)	
Asian		216 (2.2)	23 (2.0)	6 (1.2)	4 (1.5)	3 (1.9)	0 (0)	
Other		986 (10)	132 (12)	60 (12)	30 (11)	24 (15)	15 (11)	
Unknown		2	0	0	0	0	0	
**Poverty: n (%)**	11,846							< 0.001
No Indicators		7,726 (80)	854 (76)	353 (72)	169 (65)	100 (63)	70 (50)	
One Indicator		845 (8.7)	113 (10)	42 (8.6)	25 (9.5)	26 (16)	20 (14)	
Two or More Indicators		1,102 (11)	158 (14)	93 (19)	68 (26)	32 (20)	50 (36)	
Unknown		17	3	0	0	0	1	
**Combined Family Income: n (%)**	10,852							< 0.001
Less than 50 K		2,527 (29)	291 (28)	163 (36)	116 (48)	52 (35)	71 (55)	
Between 50 K and 100 K		2,499 (28)	315 (31)	110 (25)	63 (26)	53 (36)	31 (24)	
Above 100 K		3,834 (43)	423 (41)	174 (39)	61 (25)	42 (29)	27 (21)	
Unknown		830	99	41	22	11	12	
**Parental Education: n (%)**	11,853							< 0.001
High School or Less		1,144 (12)	117 (10)	57 (12)	36 (14)	17 (11)	22 (16)	
Post-Secondary but No College		2,710 (28)	321 (29)	143 (29)	107 (41)	63 (40)	60 (43)	
Undergraduate or Above		5,826 (60)	688 (61)	287 (59)	119 (45)	77 (49)	59 (42)	
Unknown		10	2	1	0	1	0	
**Birth Weight (oz): Mean (SD)**	11,353	112 (23)	111 (24)	112 (23)	109 (23)	112 (26)	112 (22)	0.32
Unknown		412	53	15	12	12	10	
**Number of Weeks Premature: Mean (SD)**	11,840	0.89 (2.15)	0.99 (2.31)	0.89 (2.20)	1.06 (2.24)	1.03 (2.44)	1.11 (2.47)	0.51
Unknown		23	0	3	0	1	0	
**Ever Sipped Alcohol: n (%)**	11,582							< 0.001
No		7,231 (77)	730 (65)	345 (72)	166 (63)	105 (67)	121 (89)	
Yes		2,203 (23)	386 (35)	133 (28)	96 (37)	51 (33)	15 (11)	
Unknown		256	12	10	0	2	5	
**Ever Drank a Full Alcoholic Drink: n (%)**	9,892							0.15
No		7,970 (100)	976 (99)	426 (100)	230 (100)	139 (99)	109 (99)	
Yes		29 (0.4)	9 (0.9)	2 (0.5)	0 (0)	1 (0.7)	1 (0.9)	
Unknown		1,691	143	60	32	18	31	
**Ever Sipped or Drank a Full Alcoholic Drink: n (%)**	11,583							< 0.001
No		7,227 (77)	729 (65)	344 (72)	166 (63)	105 (67)	121 (89)	
Yes		2,208 (23)	387 (35)	134 (28)	96 (37)	51 (33)	15 (11)	
Unknown		255	12	10	0	2	5	

Standard Deviation (SD). Child Behavior Checklist (CBCL). Diagnostic and Statistical Manual of Mental Disorders, 5th Edition (DSM5). *Pearson’s Chi-squared test for categorical measures; Kruskal-Wallis rank sum test for continuous measures.

### Regression results for global brain volume measures

We sought to determine whether developmental trajectories for total brain volume measures between 10 and 12 years of age differed between those who developed SB from those who did not. Accordingly, we applied a linear mixed effects regression approach with the primary aim of characterizing developmental changes in brain volume over time (i.e., the main effects of *timepoint*) and assessing whether developing SB moderated these developmental changes (i.e., the interaction effect between *developing SB* and *timepoint*). The robustness of these associations was assessed by including additional covariates in regression models in a stepwise approach.

**Fig. 1:**
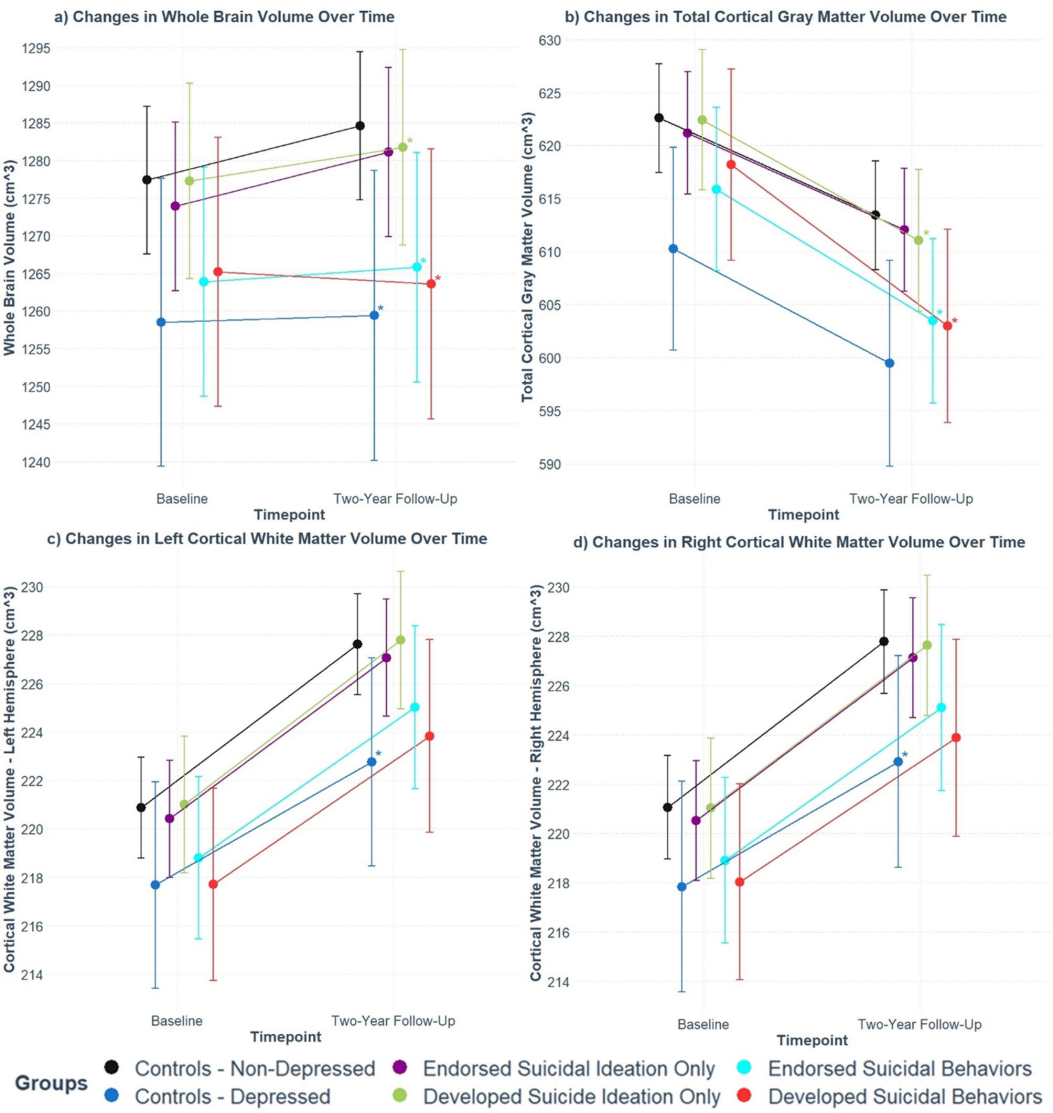
Developmental Trajectories for (a) Total Brain Volume, (b) Total Cortical Gray Matter Volume, (c) Total Left Cortical White Matter Volume, and (d) Total Right Cortical White Matter Volume. Error bars represent 95% confidence intervals for the estimated marginal means. Regressions were corrected for age, sex, self-reported race/ethnicity, and SES factors (combined family income, highest parental education, and impact of poverty). Asterisks (*) indicate groups with significantly different *group* by *timepoint* interaction effects, relative to the non-depressed control group (p.adjusted < 0.05).

For total brain volume (which includes cortical white, cortical gray, cerebellar, and subcortical volumes), we found positive main effect of *timepoint* suggesting significant rates of total brain growth (by about 7 cm^3^ in the non-depressed control group (Supplementary Table 1). We also found significant negative *group* by *timepoint* interaction effects for depressed controls, those who developed SI only, those who endorsed SB, and those who developed SB, suggesting diminished rates of growth for all these groups compared to non-depressed controls (Fig. 1a). These effects remained significant even after including race/ethnicity and SES factors as covariates in the regression models (Supplementary Tables 1 and 2).

To directly compare the rates of growth between those who developed SB and the other groups with significant *group* by *timepoint* interaction effects in the full regression models (with sex, age, race/ethnicity, and SES indicators as covariates), we applied several general linear hypotheses post-hoc tests. We found that those who developed SB exhibited significantly more diminished rates of total brain volume growth compared to those who developed SI only, but no significant differences compared to those who ever endorsed SB and depressed controls (Supplementary Table 3), indicating a lack of specificity. Notably, those who developed SB exhibited significantly lower total brain volumes compared to non-depressed controls at the two-year follow-up timepoint (Supplementary Table 4). To account for differences in total brain volume at birth, we subsequently included birth weight (adjusted for weeks of prematurity) as a covariate in our regressions as a proxy for neonatal total brain volume. All the significant effects of interest remained, although slightly attenuated (Supplementary Table 5).

Total brain volume growth reflects the net developmental changes in the brain’s gray, white, cerebellar, and subcortical matter volumes. Therefore, we examined each of these volumes separately to determine if differing developmental trajectories in any of these volumetric components were driving the differences in total brain development.

For total cortical gray matter volume, we found a significant negative main effect of *timepoint*, indicating overall reductions in total cortical gray matter by about 9 cm^3^ during this developmental window in non-depressed controls (Supplementary Table 1). Significant negative *group* by *timepoint* interaction effects were found between those who developed SI only, endorsed SB, and developed SB, even after adjusting for covariates (Supplementary Table 2). These findings suggest significantly steeper rates of cortical gray matter shrinkage in these groups compared to non-depressed controls (Fig. 1b). Post-hoc tests revealed those who developed SB exhibited significantly steeper rates of cortical gray matter shrinkage than those who developed SI only, but not compared to those who had ever endorsed SB (i.e. those in which it was unclear whether they had developed, maintained, or possibly recovered from SB) (Supplementary Table 3). In those who developed SB, cortical gray matter volume decreased by approximately 15 cm^3^ (Supplementary Table 6), and was significantly lower compared to non-depressed controls by two-year follow-up (Supplementary Table 4)).

To account for any reductions in cortical gray matter volume due to alcohol consumption, we included ever-sipping alcohol or ever-finishing a full alcoholic drink as a covariate in our regressions. The regression effects of interest were not substantially impacted (Supplementary Table 7).

In the ABCD study data, right and left cortical white matter volumes individually, but not total cortical white matter volume, were available. For both total left and right cortical white matter volumes, we found significant positive main effects of *timepoint* indicating growth (by about 7 cm^3^ on each side) in these brain volumes in non-depressed controls (Supplementary Table 1). Furthermore, a significant negative *group* by *timepoint* interaction was found for depressed controls only (Supplementary Table 2), indicating diminished rates of total left and right cortical white matter growth in this group (Fig. 1c and d). Post-hoc tests showed that depressed controls still exhibited significant rates of growth, though at a lower rate of approximately 5 cm^3^ for each hemisphere across the two years of this study (Supplementary Table 4).

For most cerebellar gray and white matter volumes, no developmental trajectories were found to be specifically altered in those who developed SB, though both depressed controls and those who developed SB exhibited similar levels of reduced left cerebellar cortex growth compared to non-depressed controls (Supplementary Fig. 1, panel a-d). Similarly, both those who developed SB and depressed controls also exhibited reduced growth for total subcortical gray matter volume (Supplementary Fig. 1, panel e), though the rates were not significantly different from each other (Supplementary Table 3).

### Regression results for psychiatric and behavioral measures

Next, we sought to identify psychiatric/behavioral measures associated with the development of SB. Similar to the univariate regressions for brain volumes, we applied linear mixed model regressions in order to characterize the developmental changes in psychiatric and behavioral measures over time (i.e., the main effect of *timepoint*) and assessing whether developing SB moderated these developmental changes (i.e., the interaction effect between *developing SB* and *timepoint*). The robustness of these associations was assessed by including additional covariates in a stepwise fashion in the regression models.

After controlling for sex, age (in months at baseline), self-reported race/ethnicity, and SES factors as covariates, we found seven psychiatric and behavioral measures that were still associated with significant positive *group* by *timepoint* interaction effects for those who developed SB at the adjusted p-value level (Table 2). Three of these measures were Child-Behavior-Checklist Measures (CBCL) scores, three were UPPS-P impulsivity scores, and one was a Behavioral Inhibition/Behavioral Activation Systems (BIS/BAS) score.

**Table 2:** Group by timepoint interaction effects in individuals who developed SB, representing differences in behavioral and psychopathological development compared to non-depressed controls. Interaction effects from multiple linear mixed effects regressions for 32 psychiatric and behavioral measures are shown. Sex, age, race/ethnicity, and SES indicators were included as covariates in the regressions.

Psychiatric/Behavioral Measures	Interaction Effect (Developed SB by Timepoint)	Std. Error	t value	*p*-value	*p*.adjusted	Significance
BIS/BAS: BIS Sum Score	0.21	0.11	1.95	0.05	0.09	
BIS/BAS: BIS Sum Score Modified	0.21	0.11	1.89	0.06	0.10	
BIS/BAS: Drive	0.12	0.11	1.09	0.27	0.36	
BIS/BAS: Drive Modified	0.12	0.11	1.09	0.27	0.36	
BIS/BAS: Fun Seeking	0.25	0.11	2.27	0.02	0.04	*
BIS/BAS: Reward Responsiveness	0.00	0.11	−0.02	0.99	0.99	
BIS/BAS: Reward Responsiveness Modified	−0.01	0.11	−0.09	0.93	0.95	
CBCL: Aggressive Syndrome	0.05	0.03	1.40	0.16	0.23	
CBCL: Anxious Depression Syndrome	0.10	0.04	2.29	0.02	0.04	*
CBCL: Attention Problems	0.03	0.04	0.61	0.54	0.63	
CBCL: Depression Syndrome	0.09	0.04	2.44	0.01	0.03	*
CBCL: DSM5 ADHD	0.02	0.05	0.50	0.61	0.70	
CBCL: DSM5 Anxiety Disorder	0.05	0.05	1.03	0.30	0.39	
CBCL: DSM5 Conduct	0.03	0.03	1.09	0.27	0.36	
CBCL: DSM5 Depression	0.15	0.04	4.27	0.00	0.00	*
CBCL: DSM5 Oppositional Defiance	0.08	0.05	1.49	0.14	0.20	
CBCL: DSM5 Somatic Problems	0.01	0.05	0.16	0.87	0.90	
CBCL: Externalizing	0.05	0.03	1.48	0.14	0.21	
CBCL: Internalizing	0.10	0.05	2.09	0.04	0.06	
CBCL: OCD	0.07	0.04	1.72	0.09	0.13	
CBCL: Rule-Breaking	0.05	0.03	1.39	0.16	0.23	
CBCL: Sluggish Cognitive Tempo	−0.04	0.03	−1.28	0.20	0.28	
CBCL: Social Problems	0.00	0.04	0.11	0.91	0.93	
CBCL: Somatic Syndrome	0.03	0.04	0.64	0.52	0.61	
CBCL: Stress	0.08	0.05	1.66	0.10	0.15	
CBCL: Thought Problems	0.07	0.04	1.87	0.06	0.10	
CBCL: Total Problems	0.07	0.06	1.17	0.24	0.32	
UPPS: Lack of Perseverance	0.31	0.11	2.93	0.00	0.01	*
UPPS: Lack of Planning	0.41	0.11	3.76	0.00	0.00	*
UPPS: Negative Urgency	0.27	0.11	2.38	0.02	0.03	*
UPPS: Positive Urgency	0.16	0.11	1.48	0.14	0.20	
UPPS: Sensation Seeking	0.13	0.11	1.26	0.21	0.28	

Asterisks (*) indicate *p*.adjusted values less than 0.05.

For the three CBCL measures, we found significant positive main effects of *timepoint* for DSM5-Depression and Depression-Syndrome scores, indicating increases in these measures over time in non-depressed controls. However, a negative main effect of *timepoint* was found for Anxious-Depression scores, indicating a decrease in this measure over time in non-depressed controls (Supplementary Table 8). Note that the Diagnostic and Statistical Manual for Mental Disorders, 5th edition (DSM5)-Depression scores were designed to more closely reflect DSM5 diagnostic criteria while Depression-Syndrome scores were derived empirically without consideration of diagnostic categories^19^. The positive *group* by *timepoint* interaction effect for those who developed SB suggested there was a rise in the rate by which the DSM5-Depression and Depression Syndrome scales were increasing, and a reduction in the rate by which Anxious-Depression scores were decreasing, over time.

**Fig. 2:**
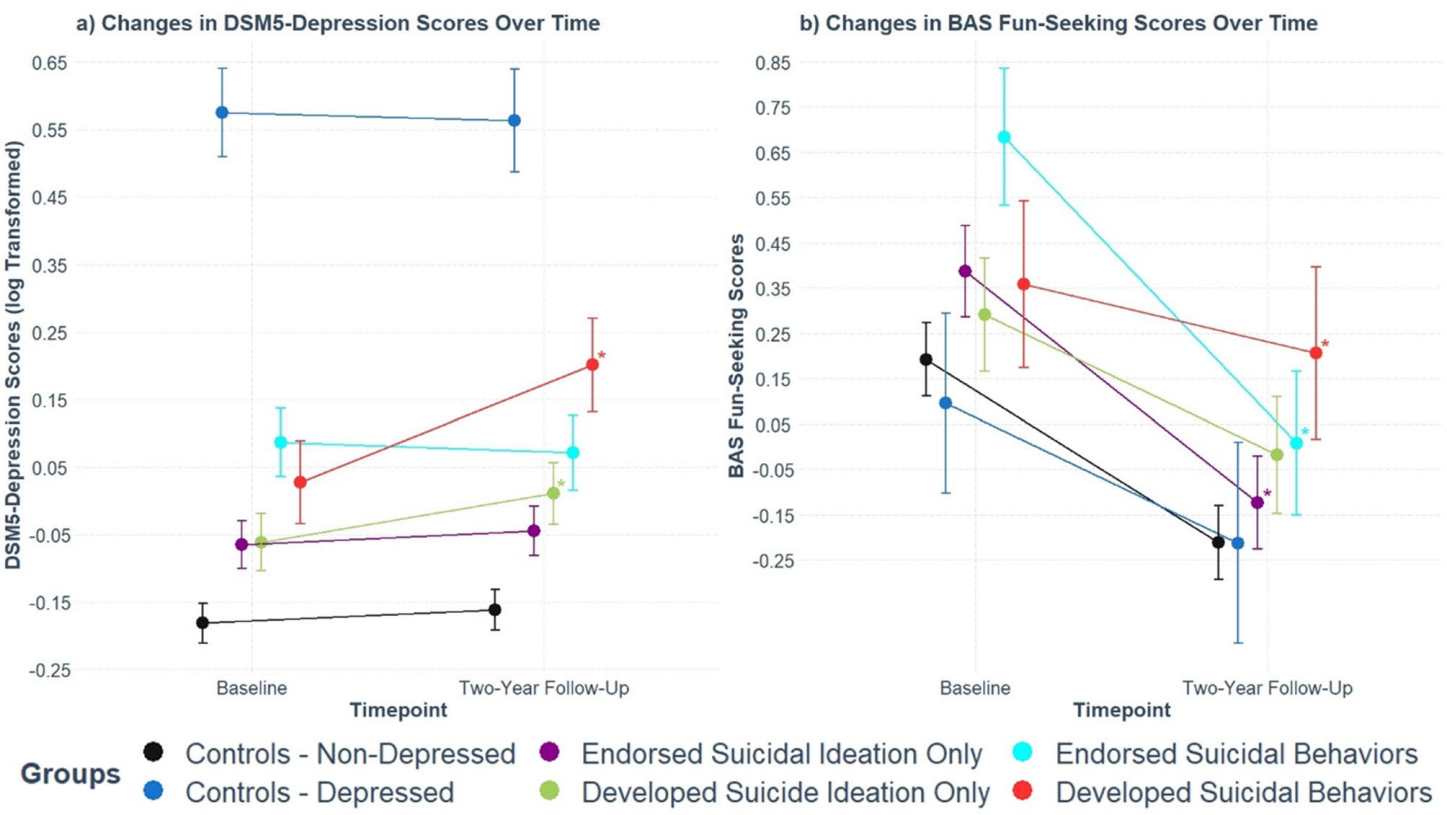
Developmental trajectories for (a) DSM5 Depression and (b) BAS Fun-Seeking Scores. Error bars represent 95% confidence intervals for the estimated marginal means. Regressions were corrected for age, sex, self-reported race/ethnicity, and SES factors (combined family income, highest parental education, and impact of poverty). Asterisks (*) indicate groups with significantly different *group* by *timepoint* interaction effects, relative to the non-depressed control group (p.adjusted < 0.05).

However, significant positive *group* by *timepoint* interaction effects were also found in those who developed SI only for all three CBCL scores (Supplementary Table 9). To directly compare the *group* by *timepoint* interaction effects between those who developed SB and those who developed SI only, we applied several post-hoc tests. We found that for those who developed SB, only the DSM5-Depression scores were associated with significantly larger positive interaction effects compared to those who developed SI only (Supplementary Table 10), suggesting those who developed SB exhibited the steepest increase in DSM5-Depression scores compared to all other groups (Fig. 2a). For Anxious-Depression and Depression-Syndrome scores, no significant differences in the *group* by *timepoint* interaction effects were found between those who developed SB and those who developed SI (Supplementary Table 10) indicating non-specific developmental changes in these measures (Supplementary Fig. 2a and 2b).

For the three UPPS-P impulsivity measures with significant *group* by *timepoint* interaction effects in those who developed SB, we also found significant positive *group* by *timepoint* interaction effects in those who developed SI only (Supplementary Table 9). However, post-hoc tests revealed they were not significantly different from the positive *group* by *timepoint* interaction effects for those who developed SB (Supplementary Table 10). These results indicate a lack of specificity in the altered developmental trajectories for UPPS-P measures (Supplementary Fig. 2c, d, and e), though those who developed SI only or SB exhibited some of the highest levels of UPPS-P measures at two-year follow-up (Supplementary Table 11).

The single BIS/BAS measure with a significant *group* by *timepoint* interaction effect for those who developed SB was the Fun-Seeking score. We found a significant negative main effect of *timepoint* for the Fun-Seeking score (Supplementary Table 8), indicating a decrease in Fun-Seeking behavior over time in non-depressed controls. Furthermore, the significant positive *group* by *timepoint* interaction effect in those who developed SB suggests the Fun-Seeking scores declined less rapidly in this group (Supplementary Table 9). Post-hoc tests revealed no significant total effect of *timepoint* in those who developed SB (Supplementary Table 12) indicating no significant estimated developmental change in BAS Fun-Seeking scores in this group specifically. Post-hoc tests also showed those who developed SB exhibited one of the highest BAS Fun-Seeking scores at two-year follow-up (Supplementary Table 11), which was not the case at baseline (Supplementary Table 11). Altogether, these results suggest that while other groups showed decreased BAS Fun-Seeking scores over time, those who developed SB exhibited preserved levels of BAS Fun-Seeking behavior over time such that their scores were among the highest at two-year follow-up (Fig. 2b).

Interestingly, significant negative *group* by *timepoint* interaction effects were found in those who ever endorsed SB or SI only (i.e. individuals in which it was unclear if they had developed, maintained, or even recovered from SB or SI only based on their pattern of responses across time) (Supplementary Table 9), suggesting that Fun-Seeking behaviors were declining at an even greater rate in these two groups compared to non-depressed controls.

Finally, although BIS summary scores were not associated with a significant *group* by *timepoint* interaction effect in those who developed SB, we sought to explore whether these scores were elevated in those who developed SB. Indeed, we found that at both baseline and two-year follow-up, those who developed SB had some of the highest levels of BIS sum scores (Supplementary Table 11). We found a significant negative main effect of *timepoint* (Supplementary Table 8) indicating a decrease in BIS sum scores in non-depressed controls over time. A significant positive *group* by *timepoint* interaction effect was only found for those who developed SI only (Supplementary Table 9). Post-hoc tests further found the overall effect of *timepoint* in those who developed SI was not significantly different from 0 (Supplementary Table 12). Altogether, the results suggest that high levels of BIS sum scores preceded the development of SB and were maintained in those who developed SI only (Supplementary Fig. 2f).

### Cross-Lagged-Panel modelling results

Since we found that Total Brain Cortical Gray Matter Volume, DSM5-Depression and BAS Fun-Seeking measures exhibited altered developmental trajectories specifically associated with SB, we next assessed whether these measures were potentially causally related. In the full ABCD sample, we first residualized these variables by regressing out the fixed effects of sex, interview age, self-reported race/ethnicity, SES factors, and the random effects of collection site and family-ID at each timepoint. Then, to test for potential causal relationships between the residualized measures, we applied a Cross-Lagged-Panel-Modelling (CLPM) approach to the full ABCD sample.

**Fig. 3:**
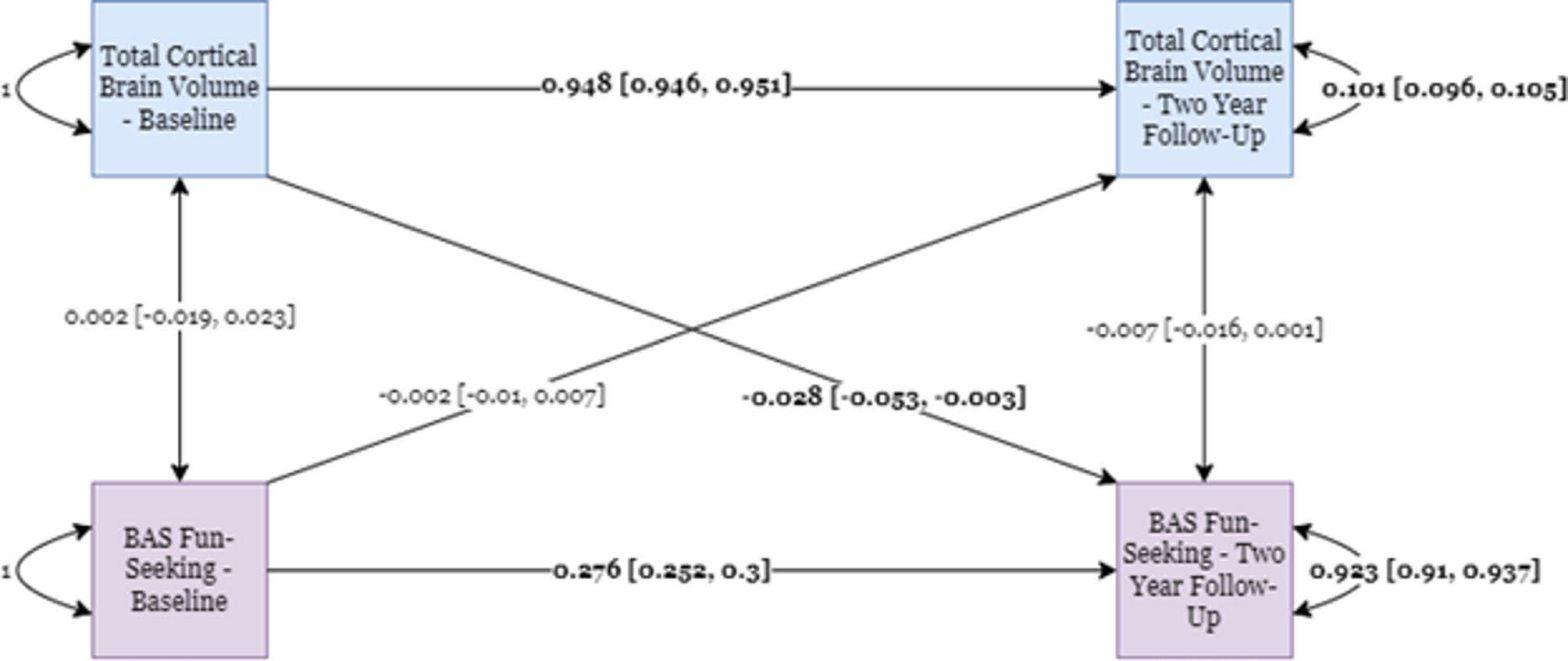
CLPM for BAS Fun-Seeking and Total Cortical Gray Matter Volume Measures. Standardized parameter estimates are shown with 95% confidence intervals adjacent in the square brackets. Statistically significant parameter estimates are bolded.

Here, we first modeled the causal relationships between the residualized BAS Fun-Seeking scores and Total Brain Cortical Gray Matter Volume measure, where single-headed arrows represent regression estimates, double-headed arrows between variables represent covariances, and double-headed arrows pointing to the same variable represents its variance at baseline or residual variance at two-year follow-up (Fig. 3).

In the CLPM model for BAS Fun-Seeking behaviors and Total Brain Cortical Gray Matter Volume, both the auto-regressive paths for BAS Fun-Seeking scores and Total Brain Cortical Gray Matter Volume were statistically significant though the larger estimate for the latter suggests that Total Brain Cortical Gray Matter Volume at baseline strongly predicts Total Brain Cortical Gray Matter Volume at two-year follow-up. Notably, there was a small, though statistically significant, negative cross-lagged path going from Total Brain Cortical Gray Matter Volume to BAS Fun-Seeking behavior, but not the other way around. When we drop the cross-lagged path going from Total Brain Cortical Gray Matter Volume to BAS Fun-Seeking behavior, the resulting model fits significantly worse than the original full model. When we drop the cross-lagged path from BAS Fun-Seeking to Total Brain Cortical Gray Matter, the model fit does not significantly deteriorate. Together, these results suggest a causal effect of Total Brain Cortical Gray Matter Volume on Fun-Seeking behaviors, but not the reverse. (Supplementary Table 13).

**Fig. 4:**
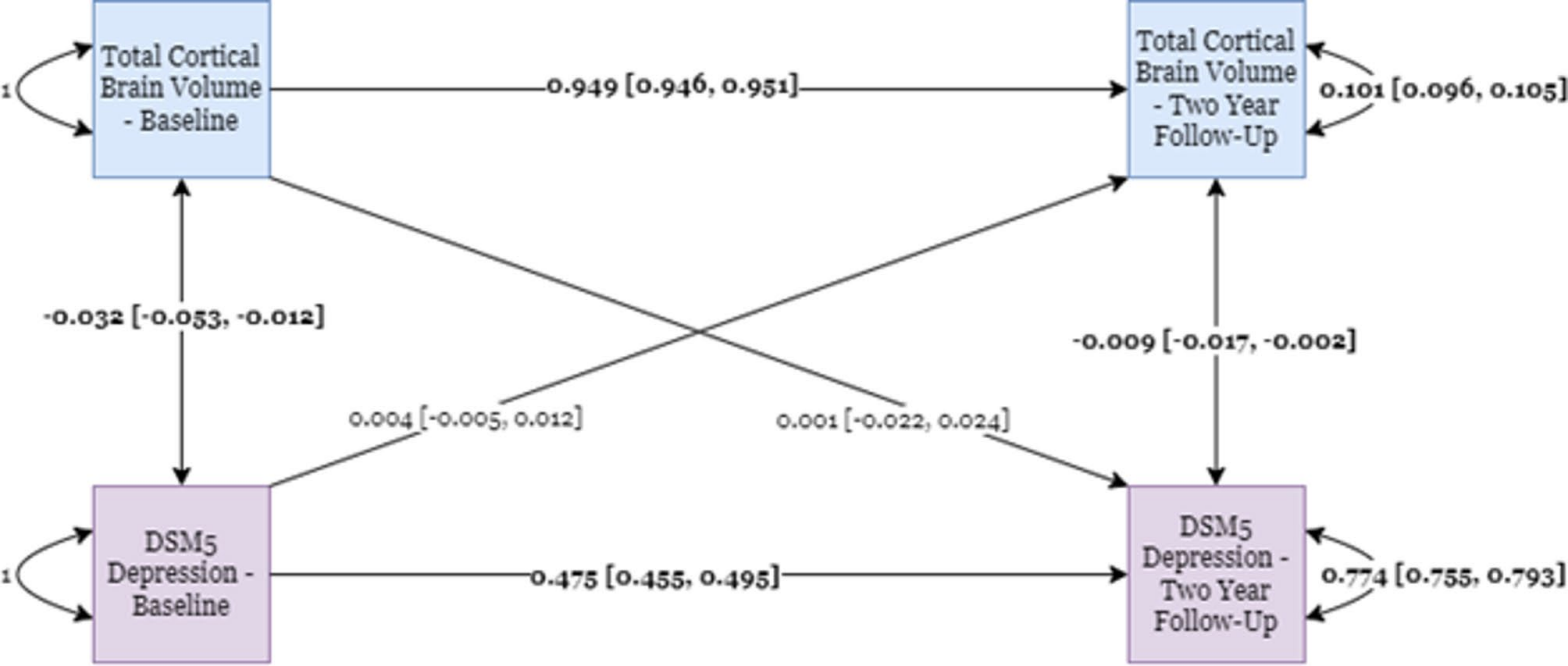
CLPM for DSM5 Depression and Total Cortical Gray Matter Volume Measures. Standardized parameter estimates are shown with 95% confidence intervals adjacent in the square brackets. Statistically significant parameter estimates are bolded.

Next, we applied a CLPM to DSM5-depression scores and Total Brain Cortical Gray Matter Volume but found no significant causal cross-lagged effects (Fig. 4). Dropping either of the cross-lagged paths in this model did not significantly worsen the model fit, suggesting a lack of any potential causal effects (Supplementary Table 14). However, there were small, but significant negative covariances between DSM5-Depression scores and Total Brain Cortical Gray Matter Volume at both the baseline and two-year follow-up timepoints. The presence of significant covariances at each timepoint but no significant cross-lagged paths potentially suggest the two years between measurements may be too long to capture any causal effects between them.

## Discussion

In our study, stunted whole brain growth was found in those who developed, or ever endorsed, SB as well as in the depressed control group, all three of which exhibited some of the lowest total brain volumes at two-year follow-up, even after accounting for brain volume at birth. Notably, lower total brain volume and surface area have been previously found to be associated with higher levels of general psychopathology in youths^20^. Thus, diminished total brain growth does not specifically distinguish youths reporting SB from depressed youths without a history of STBs.

However, we found that those reporting SB exhibited the steepest reductions in total cortical gray matter volume. Accelerated reductions in cortical gray matter volumes have also been associated with binge-drinking during adolescence^21^. However, the prevalence of alcohol use was very low in the ABCD sample (Table 1). Nevertheless, we included ever-sipping or ever-drinking a full alcohol beverage as a covariate in our regressions, though none of the effects of interest were substantially affected (Supplementary Table 7). These results suggest the accelerated reductions in cortical gray matter specifically associated with SB were not driven by alcohol use.

Interestingly, binge-drinking has been associated with higher levels of cortisol, a hormone produced in response to stress which can have excitotoxic effects on the brain^22^. Low socioeconomic status (SES) may indicate significant environmental stress and indeed, those reporting SB in the ABCD study had some of the lowest SES indicators (Table 1). We found that higher combined family income and parental education were associated with larger brain structure volumes while higher poverty scores were associated with smaller brain structure volumes for all brain volume measures (Supplementary Table 15). However, low SES factors were also found in the depressed control group who have never reported any STB, suggesting low SES was not specific to individuals reporting SB.

Instead, the psychiatric and behavioral measures that did distinguish those who developed SB from other groups were the significantly steeper increases in DSM5-depression scores and significantly slower decline in Behavioral Activation System (BAS) Fun-Seeking scores over time. Importantly, it was the increased *rate of change* in DSM5-depression, not its absolute levels, that were found specifically in those who developed SB. While depression is related to several risk factors and processes leading to suicide ideation and desire (such as hopelessness and reduced connectedness^5^ in ideation-to-action frameworks for suicide, we found that those who developed SB exhibited significantly lower absolute levels of depression than the depressed control group without any history of STB. Our findings suggest that high levels of depression per se are not sufficient to lead to SI or SB, but rather it is the dynamic change in depression levels that may significantly impact suicide risk. While increases in depression across a two-year period may not necessarily be considered an acute or precipitating risk factor for suicide, it may impact cognitive, emotional, physiological, and motivational/behavioral systems to increase the baseline risk for suicide and propensity for activation of the *suicidal mode*, as described in the FVT of suicide^6^.

Using our CLPM approach, we found that lower total cortical gray matter volume at baseline predicted higher levels of BAS Fun-Seeking behaviors at two-year follow-up, but not the other way around, suggestive of a unidirectional causal process. Furthermore, those who developed SB exhibited some of the highest levels of both BAS Fun-Seeking and Behavioral Inhibition System (BIS) summary scores at two-year follow-up. Our findings are consistent with a previous study of over 10,000 adults which found that individuals exhibiting both high BIS and BAS sensitivities were at the highest risk for past-month suicide attempt^23^. It was argued that SB may be an avoidance-based escape strategy wherein high levels of BIS sensitivity in response to intense emotional strain may in turn increase BAS sensitivity that inflates the value of relief from escaping those aversive stressors thereby increasing the motivation to engage in SB. Our results show that high levels of BIS sensitivity precede the development of SB and that developmentally inappropriate maintenance of higher levels BAS-Fun-Seeking distinguish those who develop SB from other groups.

In contrast to those with a history of SB, depressed non-suicidal adults have been found to exhibit high levels of BIS but low levels of BAS^24^. Another study in non-depressed college students found that natural and induced sad mood also led to reduced BAS and thus, decreased reward sensitivity^25^. Thus, concurrently high BIS and high BAS may be useful markers to distinguish between depressed but non-suicidal individuals from those at significantly elevated risk of suicide attempts.

Our CLPM approach was limited to specifying models testing the relationships between continuous brain and behavioral measures without directly assessing their predictive effects on the risk of STB. While alternative CLPM approaches using binary or ordinal measures have been developed^26^more dimensional measures of suicidality, such as the suicide capability scale^27^may be more effective at capturing the severity of suicidality and better suited for statistical analyses exploring the relationship between suicidality, brain based measures, and behavioral risk factors. Furthermore, CLPM approaches do not model any developmental growth factors representing brain or behavioral developmental trajectories. Given the findings of our study, it would be of interest to specify more integrative models testing whether developmental changes in depression and total cortical gray matter volume are predictive of SB as well as any bi-directional or even three-way relationships between these measures. These complex relationships and developmental processes may be modeled using more sophisticated structural equation modelling approaches such as latent growth curve (LGC)^28^ and latent variable-autoregressive latent trajectory models^29^. However, modeling developmental growth requires repeated measures from at least 3 timepoints^28^which may be made available in future data releases form the ABCD study.

We acknowledge that while alterations in total brain gray matter volume development may be significant markers of suicide risk, additional investigation of specific brain regions are required to further characterize the mechanisms underlying SI and SB. For example, one study found that age was significantly positively correlated with cortical thickness in the temporal cortices and the right insula, as well as with right putamen volume, specifically in adolescents with a history of suicide attempt^30^. These findings suggest that altered maturation in specific brain regions may more precisely characterize the specific neurocircuitry underlying suicide behaviors during adolescence. Furthermore, other studies have focused on specific brain regions previously shown to be implicated in STB and rumination^31^ as well as brain regions exhibiting lower gray matter volume in mood disorders in which altered expression of genes associated with immune and neurodevelopmental processes between high vs. low suicide mortality risk groups were found^32^. Other factors that may impact brain structure and behavioral development include metabolic and nutritional factors that influence individual differences in weight, height, and Body-Mass-Index and are deserving of investigation in future studies. Pubertal development may also be an important factor to consider as it is associated with brain maturation^33^ and some studies have found mediation effects of accelerated pubertal development on the association between the stressful family environments and altered brain structure^34^. A similar approach may be taken in futures studies to investigate whether pubertal development may mediate the associations between altered brain development and risk factors for suicide behaviors.

Importantly, future studies will also need to investigate how altered brain structure related to SB may impact brain function. Recent studies in adults with a history of suicide attempt have found decreased frontoparietal network activity and connectivity^35^ as well as decreased connectivity in several insula to cortical networks which were correlated with increased psychological pain avoidance and loss aversion^36^. Whether similar alterations in brain functional connectivity distinguish adolescents with SB from those with SI will be an important avenue of investigation.

We did not focus on the findings from the depressed-control group although they exhibited distinct developmental trajectories in cortical white brain matter volume and some of the lowest brain volumes in the study. We note that in addition to clinically relevant levels of depression symptomatology, this group also exhibited some of the highest levels of internalizing, externalizing, and total problem scores (Table 1). Importantly, we found that these individuals already exhibited significantly high levels of depression symptomatology at baseline suggesting that their psychopathology initiated before the start of the study. It is possible that significant stressors experienced earlier in life, or certain genetic predispositions, may have caused both earlier development of psychopathology and earlier development of altered brain growth trajectories. Thus, the relative lack of reduction in cortical gray matter volume in this group may reflect floor effects of significant reductions in brain volume. How these differences in brain structure development contribute to altered brain function and psychopathology remains an important area of investigation and critical to understanding how they are distinct from the processes underlying SB.

Finally, while this study focused on the development of SB in early adolescence, there was a small group of 22 individuals who appeared to have attempted suicide at an earlier age than at baseline (or starting at baseline) (Supplementary Table 19) and who consistently reported this history across follow-up. These individuals may represent a distinct group with a unique liability to SB, given their earlier presentation, and for whom SB may have been “maintained” across the study timepoints. However, they were amalgamated into the “endorsed SB” group and not excluded from the study to avoid introducing any exclusion bias. We acknowledge the significant heterogeneity within this group and the importance of future studies to differentiate subgroups within it to better characterize the potentially unique neurological and behavioral processes associated with periods of symptom maintenance, deterioration, or recovery from SB.

In conclusion, our study found significant differences in total cortical gray matter volume development specifically in those who reported SB, as well as altered trajectories in depression and BAS-fun-seeking behaviors that distinguished those who developed SB from those reporting SI only, and depressed and non-depressed controls. A possible causal relationship between total cortical gray matter volume and BAS-fun-seeking behaviors was also identified with CLPM modelling. Our study’s strengths include its large and nationally representative sample size of children in late childhood/early adolescence as well as its use of longitudinal brain imaging and behavioral/psychiatric data allowing for the study of developmental markers specifically associated with the emergence of SB. Our study was limited by the lack of focus on specific brain regions or neurocircuits that may provide greater mechanistic insight into the causes of SB. Furthermore, we did not distinguish between self-reported preparatory actions toward SB, interrupted and aborted attempts, and actual attempts which may correspond to distinct stages or aspects of ideation-to-action frameworks of suicide^37^ or reflect differences in suicide liability driven by unique developmental and neurobiological processes. We also acknowledge that our findings characterize non-lethal SB and not suicide death, which is less common in this age group^38^. Future studies addressing this heterogeneity in SB and using more integrative modeling approaches will be useful for further characterizing the complex relationships between the brain, behaviors/psychopathology, and suicide risk.

### Methods

#### Participant sample

The Adolescent Brain Cognitive Development (ABCD) Study is a longitudinal study of brain and behavioral development consisting of 11,878 youths recruited across 21 sites across the USA. Participants were recruited when they were 9–10 years old. Details of the study collection information are reported elsewhere^39,40^. We used the ABCD study 4.0 data release (DOI:10.15154/1523041) which contained longitudinal data collected up to 2-year follow-up.

### Measures

#### Suicidal thoughts and behaviors (STBs)

Youth self-reported suicidal thoughts and behaviors (STB) items from the Kiddie-Schedule for Affective Disorders and Schizophrenia (KSADS) questionnaire (data structure: abcd_ksad501) were used to identify individuals who reported either current or past questionnaire items related to STB at any of the following three time-points when the data were collected: baseline, 1-year follow-up, or 2-year follow-up. We chose to use youth self-reported STB measures instead of parent reported measures because the majority of youth self-reported suicidality have been found to be unknown to or unreported by parents or care givers in the ABCD study^41^. As the prevalence of STB is already low, using youth self-reported STB would additionally maximize sample size and statistical power.

We defined suicide ideation (SI) as endorsement of any of the following 5 KSADS items: past or present (1) passive suicide ideation, (2) non-specific active suicide ideation, (3) active suicide ideation with method, (4) active suicide ideation with intent, or (5) active suicide ideation with a plan.

Similarly, we defined suicide behaviors (SB) as endorsement of any of the following 4 KSADS items: any past or present (1) preparatory actions toward imminent suicidal behavior, (2) interrupted attempt, (3) aborted attempt, or (4) suicide attempt.

We then sorted the study participants who have ever reported any STB into the following mutually-exclusive groups based on their pattern of responses across the three timepoints: (1) those who developed suicide behaviors (SB), (2) those who have ever *endorsed* SB, (3) those who developed suicide ideation (SI) only without suicide behaviors, and (4) those who have ever *endorsed* SI only without endorsing any suicide behaviors (Table 3). Note, participants classified as those who have endorsed SB represent a heterogeneous group of individuals whose endorsement of past or present SB across baseline, one-year, and two-year follow-up could not be confidently interpreted (Supplementary Table 19). The vast majority (~ 75%, *n* = 197) of this group have endorsed SB at only one time point; at either baseline or at one-year follow-up. Another ~ 16% (*n* = 43) reported SB at two timepoints in a manner inconsistent with the development of SB. The remaining 8% (*n* = 22) endorsed SB at all 3 timepoints. There are many possible reasons for these patterns of responses including forgetting, over-reporting, or symptom fluctuations that we are not able to discern. However, we included these participants in our analyses as a group assumed to exhibit relatively higher liability to SB while recognizing the limitations in interpreting results pertaining to this group due to its heterogeneity. A similar rationale was used to classify individuals who have ever endorsed SI.

**Table 3:** Classification of participants who have ever reported STB.

Timepoint	Reported Suicide Ideation	Reported Suicide Behaviors	Assigned Group
**Baseline**	Any response	No	Developed Suicide Behaviors.
**1-Year Follow-Up**	Any response	No	
**2-Year Follow-Up**	Any response	Yes	
**Baseline**	Any response	No	Developed Suicide Behaviors.
**1-Year Follow-Up**	Any response	Yes	
**2-Year Follow-Up**	Any response	Yes	
**Baseline**	Any response	Yes at any timepoint(s), but not in any of the patterns above.	Endorsed Suicide Behaviors.
**1-Year Follow-Up**	Any response		
**2-Year Follow-Up**	Any response		
**Baseline**	No	No	Developed Suicide Ideation Only.
**1-Year Follow-Up**	No	No	
**2-Year Follow-Up**	Yes	No	
**Baseline**	No	No	Developed Suicide Ideation Only.
**1-Year Follow-Up**	Yes	No	
**2-Year Follow-Up**	Yes	No	
**Baseline**	Yes at any timepoint(s), but not in any of the patterns above.	No	Endorsed Suicide Ideation Only.
**1-Year Follow-Up**		No	
**2-Year Follow-Up**		No	
**Baseline**	No	No	Controls (either Depressed or Non-Depressed)
**1-Year Follow-Up**	No	No	
**2-Year Follow-Up**	No	No	

We also assigned those who have never reported any STB but who have exhibited clinically significant levels of depression, indicated by a Child Behavior Checklist (CBCL) DSM5-Depression t-score greater than 69 (representing scores greater than the 98th percentile), to a depressed control group. Finally, we assigned individuals who have never reported STB and who have not exhibited clinically significant levels of depression to a non-depressed control group.

#### Brain volume

T1 weighted MRI brain volume images were used (data structure: abcd_smrip10201). Modality-specific imaging inclusion flags based on recommended inclusion criteria were used to filter imaging measures for inclusion in our analyses (data structure: abcd_imgincl01). Specifically, we used the T1-weighted image inclusion flag which was based on the following criteria: T1 series passed rawQC, FreeSurfer QC not failed, and Derived results exist (summarized here: https://wiki.abcdstudy.org/release-notes/imaging/quality-control.html). Detailed image acquisition protocols for structural brain MRI data have been previously documented^42^. Briefly, to ensure high quality and reliable structural brain MRI data, trained technicians assessed the severity of five categories of image artifact/reconstruction which included motion, intensity inhomogeneity, white matter underestimation, pial overestimation, and magnetic susceptibility artifact, in order to provide inclusion or exclusion recommendations. We selected 9 brain volume measures representing large total brain structures : (1) whole brain, (2) total cortical (gray matter), (3) total left cortical white matter, (4) total right cortical white matter, (5) total left cerebellar cortex, (6) total right cerebellar cortex, (7) total left cerebellar white matter, (8) total right cerebellar white matter, and (9) total subcortical gray matter brain volumes.

### Child behavior checklist (CBCL) and other behavioral measures

CBCL raw scores consisting of 20 parent reported measures of their child’s behaviors were selected for use in our analyses (data structure: abcd_cbcls01), including six DSM5-based scales. CBCL raw scores are preferred for statistical analyses because they reflect more of the natural variation that occurs in these scores that may be otherwise reduced in the standardized T-scores (DSM-Oriented Guide for the Achenbach System of Empirically Based Assessment ASEBA).

Other behavioral measures with data available at both baseline and two-year follow-up timepoints were also selected for our analyses (data structure: abcd_mhy02). These were the UPPS-P Impulsive Behavior scale scores, which includes 5 subscales based on youth self-reported responses (urgency, premeditation, perseverance, sensation-seeking, and positive urgency) to assess impulsive behavior, and the Behavioral-Inhibition and Behavioral Approach Systems Scales (BIS/BAS), which includes 4 scores based on youth self-reported responses (BIS summary score, BAS Drive, BAS Reward Responsiveness, and BAS Fun-Seeking) assessing behavioral motivation and inhibition.

Altogether, 32 psychiatric and behavioral measures were selected for our analyses. These mental health and behavioral measures from the ABCD study are reviewed extensively elsewhere^43^.

For the CBCL measures, the raw-scores were log-transformed to improve the normality of the data. No data transformations were applied to UPPS-P and BIS/BAS behavioral measures as they were already normally distributed. Normality of the data was assessed by inspecting histogram and quantile-quantile plots of regression residuals. Similarly, assumptions of homoscedasticity were assessed by inspecting regression residuals plotted against model fitted values. For brain volume measures, outliers exceeding 1.5 times the interquartile-range of values were removed prior to applying regression analyses. The outliers removed for each global brain structure measure represented on average less than 1% of all observations (Supplementary Table 16).

#### Race, ethnicity, and Socio-Economic status (SES) factors

Self-reported race/ethnicity was used as a covariate and included the following groups: White, Black, Hispanic, Asian, and Other (data structure: acspsw03).

Several SES factors were also selected, though these were assessed at 1-year follow-up (data structure: pdem02). Parental education data were concatenated into 3 levels: High-school or less, Post-Secondary Education but no College, and Undergraduate or above. If both the primary parent and partner education data were available, the higher was taken. A poverty score indicating the number of significant poverty related experiences was constructed by creating 3 levels: no significant poverty experiences, 1 significant poverty experience, and 2 + significant poverty experiences. Finally, combined yearly family income data were concatenated into 3 levels: less than $50,000, between $50,000 and $100,000, and above $100,000.

#### Alcohol use

Alcohol use and consumption behaviors were obtained from two data structures containing responses to sipping or drinking a full alcoholic drink at baseline (data structure: abcd_ysu02) and 6-month follow-up (data structure: abcd_ymypisu01). The responses were combined to create 3 lifetime alcohol use or consumption behaviors representing (1) ever taking a sip of alcohol, (2) ever drinking a full drink of beer, wine or liquor, or (3) ever taking a sip of or drinking a full alcoholic beverage. Alcohol use has been previously linked to alterations in brain structure^21^ so we controlled for alcohol use in sensitivity analyses.

### Birth weight

Birthweight and the number of weeks born premature were obtained from the developmental history data structure (data structure: dhx01). Total birthweight was converted to ounces and weeks premature was included in downstream analyses to adjust for gestational age.

### Non-Suicidal self injurious behaviors

Non-Suicidal Self Injurious (NSSI) behaviors were obtained from the KSADS questionnaire (data structure: abcd_ksad501). Past and present NSSI behaviors were combined to create a lifetime NSSI behavior measure.

### Statistical analyses

All data processing and statistical analyses were performed using the R statistical software version 4.3.1.

#### Univariate regressions

Univariate linear mixed effects regressions were applied to 9 brain volume measures and 32 behavioral measures using the *gamm4* package. The regression estimates of interest included the main effects of *timepoint*, estimating the change in a measure across 2 years of development, and the *group* by *timepoint* interaction effects for the STB groups, which estimate the differences in the developmental trajectories between the different groups defined above, relative to a reference group (see Methods: Suicidal Thoughts and Behaviors). The reference group in the regressions was the non-depressed control group while the comparison groups were the depressed controls, those who ever endorsed only SI or ever endorsed SB (with or without SI), and those who developed only SI or developed SB (with or without SI).

To directly compare the significant *group* by *timepoint* interaction effects between the comparison STB groups (instead of with just the reference group), we applied general linear hypotheses post-hoc tests, which specifies contrasts utilizing linear combinations of the respective regression parameter estimates, using the *glht()* function from the *multcomp* package^44^. For example, to compare *group* by *timepoint interaction* effects between those who developed SB and those who developed SI only, we specified a null hypothesis that the difference between those interaction effects would be 0 (ex. Developed SB by Timepoint - Developed SI Only by Timepoint = 0). A statistically significant contrast estimate (*p* < 0.05) would indicate a significant difference between the interaction effects. Additionally, we specified contrasts to test whether there were group differences between those who developed SB and the other comparison groups for the brain and psychiatric/behavioral measures of interest at each timepoint (ex. Developed SB - Developed SI Only at Baseline = 0; Developed SB - Developed SI Only at Two-Year Follow-Up = 0). Finally, we specified contrasts to test whether the total effect of *timepoint* in those who developed SB for brain and psychiatric/behavioral measures was significant by specifying a null hypothesis that the sum of the main effects of *timepoint* and the *group* by *timepoint* interaction effect for those who developed SB would be 0 (ex. Timepoint + Developed SB by Timepoint = 0). A statistically significant contrast would indicate a significant rate of change for a particular measure in those who developed SB. All post-hoc tests were adjusted for multiple comparisons with the Benjamini-Hochberg method.

Additionally, for all regressions, self-reported sex and interview age at baseline were included as covariates. Self-reported race/ethnicity and then SES factors were included sequentially to assess the impact these covariates had on the regression estimates of interest. Study collection *site*, *family-ID* nested within site, and *subject-ID* nested within family-ID were included as random effects to account for any non-independence of data and other hierarchical clustering. Intraclass Correlations (ICC) for each of the nested random effects for the full regression models including all covariates were reported in Supplementary Tables 17 and 18. We adjusted the p-values for multiple comparisons using the Benjamini-Hochberg method.

#### Cross lagged panel modelling (CLPM)

CLPM is a structural equation modeling approach used to model hypothesized causal relationships between variables measured at two or more timepoints. Here, we were interested in testing for the potential causal relationships between brain structure and psychiatric/behavioral measures associated with STB collected at the baseline and two-year follow-up timepoints. To do so, we specified CLPM models using the *OpenMx* package^45^. Briefly, CLPM tests whether one measure at an earlier time-point can predict a different measure at a later time-point, (i.e., the cross-lagged paths), while controlling for the auto-regressive relationships between the same variable measured at two timepoints, and the covariances between different variables within each timepoint. The magnitude and significance of the cross-lagged causal paths can then be assessed by computing confidence intervals for the estimated parameters of interest and by comparing the fit between models with and without them.

For the brain and psychiatric/behavioral measures of interest, we regressed out the fixed effects of sex (assigned at birth), interview age, self-reported race/ethnicity, SES factors, and the random effects of collection site and family-ID at each timepoint. For the CBCL DSM5-Depression score, we combined the residualized variables from the two timepoints into a single column and then applied the boxCox transformation to this data to improve normality. We then applied the CLPM models to the residualized and transformed data. To compare model fits, we used the mxCompare() function to apply a likelihood ratio test of the difference in the − 2log-likelihood of the nested and reference models. Twice the difference in log-likelihood between a base model and a sub-model (defined as having two or more parameters equated or fixed in the base model) is asymptotically distributed as chi-squared with degrees of freedom equal to the difference in the number of free parameters in the two models^46^.

## Supplementary Information

Below is the link to the electronic supplementary material.

Supplementary material 1 (PDF 1218.5 kb)

## Data Availability

Data used in the preparation of this article were obtained from the Adolescent Brain Cognitive Development SM (ABCD) Study (https://abcdstudy.org), held in the NIMH Data Archive (NDA). This is a multisite, longitudinal study designed to recruit more than 10,000 children age 9–10 and follow them over 10 years into early adulthood. The ABCD Study is supported by the National Institutes of Health and additional federal partners under award numbers U01DA041048, U01DA050989, U01DA051016, U01DA041022, U01DA051018, U01DA051037, U01DA050987, U01DA041174, U01DA041106, U01DA041117, U01DA041028, U01DA041134, U01DA050988, U01DA051039, U01DA041156, U01DA041025, U01DA041120, U01DA051038, U01DA041148, U01DA041093, U01DA041089, U24DA041123, U24DA041147. A full list of supporters is available at https://abcdstudy.org/federal-partners.html. A listing of participating sites and a complete listing of the study investigators can be found at https://abcdstudy.org/consortium_members/. ABCD consortium investigators designed and implemented the study and/or provided data but did not necessarily participate in the analysis or writing of this report. This manuscript reflects the views of the authors and may not reflect the opinions or views of the NIH or ABCD consortium investigators. The ABCD data repository grows and changes over time. The ABCD data used in this report came from the Curated Annual Release 4.0 - DOI:10.15154/1523041. DOIs can be found at https://nda.nih.gov/abcd/abcd-annual-releases. The scripts used for the analyses supporting this study are available at our Open Science Framework repository: https://osf.io/nxyb5/?view_only=697b4d5a983e44cb9dd021d8df77512a.
