# Supplementary material for: Adolescent suicide behaviors associate with accelerated reductions in cortical gray matter volume and slower decay of behavioral activation Fun-Seeking scores
Source: Sci Rep. 2025 Sep 25;15:32886. doi: 10.1038/s41598-025-16856-y (PMC12464210; doi:10.1038/s41598-025-16856-y)
Supplement: Supplementary file 1 — Supplementary material 1 (PDF 1218.5 kb) [file 41598_2025_16856_MOESM1_ESM.pdf]

Adolescent Suicide Behaviors Associate with Accelerated Reductions in Cortical Gray Matter Volume and Slower Decay of Behavioral Activation Fun-Seeking Scores

Yi Zhou*[1], PhD and Michael C. Neale[1], PhD

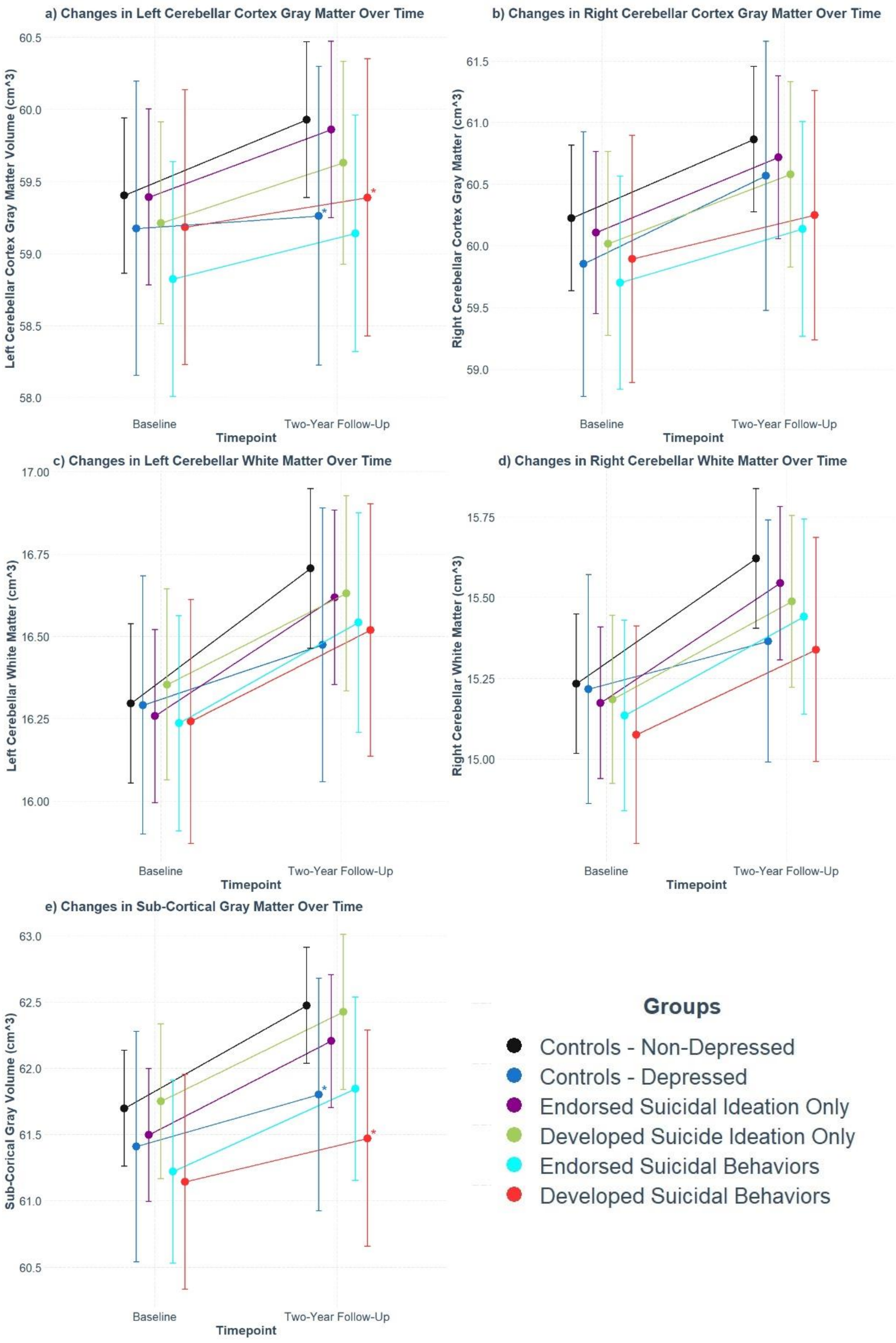

Supplementary Figure 1 - Developmental Trajectories for a) Total Left Cerebellar Cortex, b) Total Right Cerebellar Cortex, c) Total Left Cerebellar White Matter, d) Total Right Cerebellar White matter, and e) Total Subcortical Gray Matter Volumes. Error bars represent 95% confidence intervals for the estimated marginal means. Regressions were corrected for age, sex, self-reported race/ethnicity, and SES factors (combined family income, highest parental education, and impact of poverty). Asterisks (*) indicate groups with significantly different *group* by *timepoint* interaction effects, relative to the non-depressed control group (p.adjusted < 0.05).

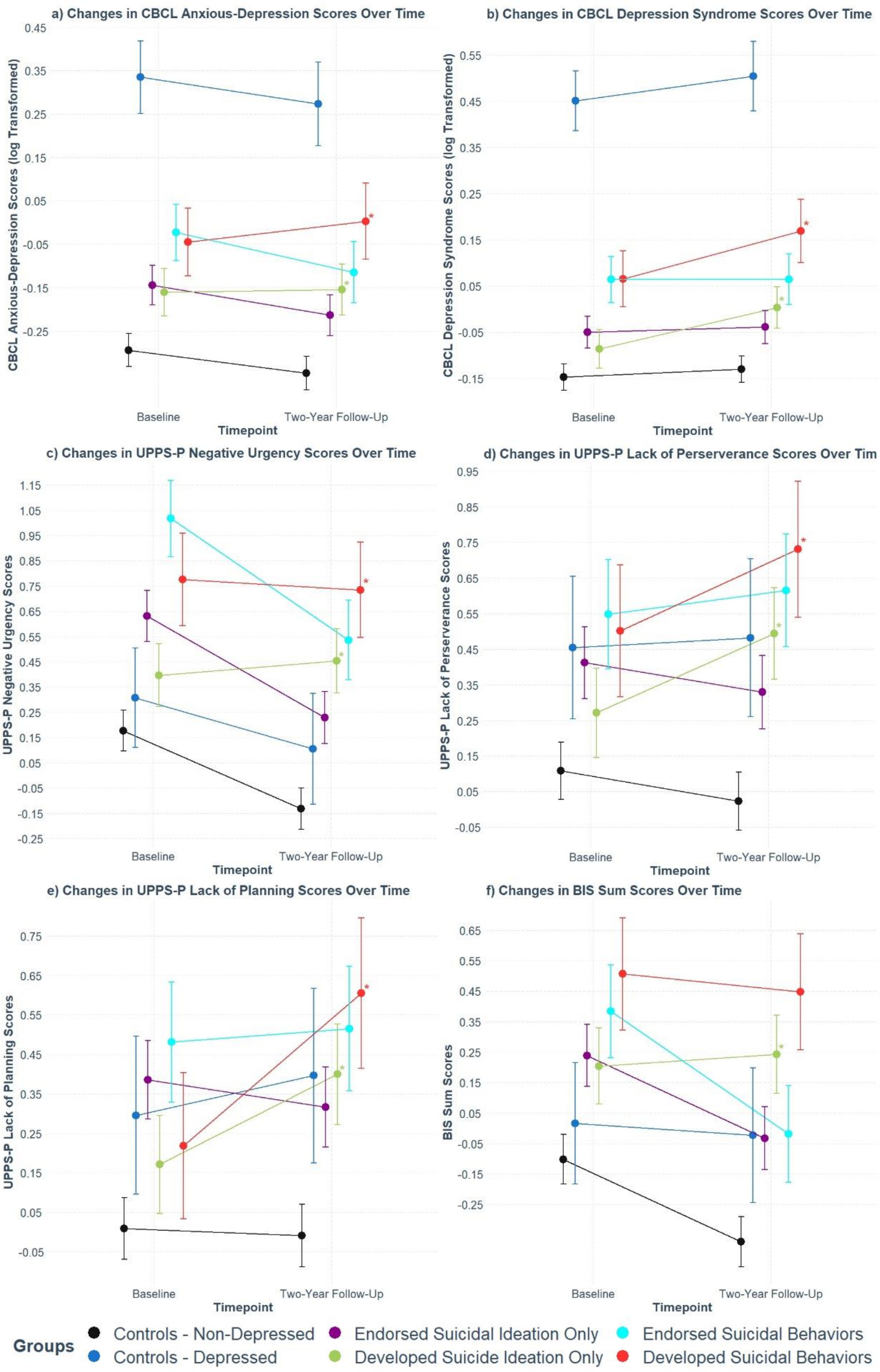
a) Changes in CBCL Anxious-Depression Scores Over Time
CBCL Anxious-Depression Scores (log Transformed)
0.45
0.35
0.25
0.15
0.05
-0.05
-0.15
-0.25
Baseline
Two-Year Follow-Up
Timepoint
b) Changes in CBCL Depression Syndrome Scores Over Time
CBCL Depression Syndrome Scores (log Transformed)
0.55
0.45
0.35
0.25
0.15
0.05
-0.05
-0.15
Baseline
Two-Year Follow-Up
Timepoint
c) Changes in UPPS-P Negative Urgency Scores Over Time
UPPS-P Negative Urgency Scores
1.15
1.05
0.95
0.85
0.75
0.65
0.55
0.45
0.35
0.25
0.15
0.05
-0.05
-0.15
-0.25
Baseline
Two-Year Follow-Up
Timepoint
d) Changes in UPPS-P Lack of Perserverance Scores Over Tim
UPPS-P Lack of Perserverance Scores
0.95
0.85
0.75
0.65
0.55
0.45
0.35
0.25
0.15
0.05
-0.05
Baseline
Two-Year Follow-Up
Timepoint
e) Changes in UPPS-P Lack of Planning Scores Over Time
UPPS-P Lack of Planning Scores
0.75
0.65
0.55
0.45
0.35
0.25
0.15
0.05
-0.05
Baseline
Two-Year Follow-Up
Timepoint
f) Changes in BIS Sum Scores Over Time
BIS Sum Scores
0.65
0.55
0.45
0.35
0.25
0.15
0.05
-0.05
-0.15
-0.25
Baseline
Two-Year Follow-Up
Timepoint
Groups
Controls - Non-Depressed
Controls - Depressed
Endorsed Suicidal Ideation Only
Developed Suicide Ideation Only
Endorsed Suicidal Behaviors
Developed Suicidal Behaviors

Supplementary Figure. 2 - Developmental trajectories for a) Anxious-Depression, b) Depression-Syndrome, c) UPPS Negative Urgency, d) UPPS Lack of Perseverance, e) Lack of Planning, and f) BIS Sum Scores. Error bars represent 95% confidence intervals for the estimated marginal means. Regressions were corrected for age, sex, self-reported race/ethnicity, and SES factors (combined family income, highest parental education, and impact of poverty). Asterisks (*) indicate groups with significantly different *group* by *timepoint* interaction effects, relative to the non-depressed control group (p.adjusted < 0.05).

Supplementary Table 1 - Main effect of *Timepoint* from multiple linear mixed effects regressions for 9 global brain volume measures representing changes in volume in the non-depressed control group between 10 and 12 years of age. Regression models with sex and age as covaraites[1]; sex, age and self-reported race/ethnicity as covariates[2]; and sex, age, race/ethnicity, and SES indicators as covaraites[3].

sMRI Measure (outcome variable)	Regression Parameter (predictor variable)	Estimate (cm^3)	Std. Error	t value	p-value	p.adjusted
Total Brain Volume[1]	Main Effect: Timepoint	7.16	0.31	22.77	0.00	0.00
Total Brain Volume[2]	Main Effect: Timepoint	7.15	0.31	22.73	0.00	0.00
Total Brain Volume[3]	Main Effect: Timepoint	7.22	0.33	22.10	0.00	0.00
Total Cortical Gray Matter Volume[1]	Main Effect: Timepoint	-9.23	0.23	-40.14	0.00	0.00
Total Cortical Gray Matter Volume[2]	Main Effect: Timepoint	-9.24	0.23	-40.24	0.00	0.00
Total Cortical Gray Matter Volume[3]	Main Effect: Timepoint	-9.19	0.24	-38.71	0.00	0.00
Total Left Cerebellar Cortex Volume[1]	Main Effect: Timepoint	0.53	0.02	21.90	0.00	0.00
Total Left Cerebellar Cortex Volume[2]	Main Effect: Timepoint	0.53	0.02	21.85	0.00	0.00
Total Left Cerebellar Cortex Volume[3]	Main Effect: Timepoint	0.53	0.02	21.13	0.00	0.00
Total Left Cerebellar White Matter Volume[1]	Main Effect: Timepoint	0.40	0.02	20.36	0.00	0.00
Total Left Cerebellar White Matter Volume[2]	Main Effect: Timepoint	0.39	0.02	20.31	0.00	0.00
Total Left Cerebellar White Matter Volume[3]	Main Effect: Timepoint	0.41	0.02	20.17	0.00	0.00
Total Left Cortical White Matter Volume[1]	Main Effect: Timepoint	6.74	0.07	92.82	0.00	0.00
Total Left Cortical White Matter Volume2	Main Effect: Timepoint	6.73	0.07	93.12	0.00	0.00
Total Left Cortical White Matter Volume[3]	Main Effect: Timepoint	6.74	0.07	90.66	0.00	0.00
Total Right Cerebellar Cortex Volume[1]	Main Effect: Timepoint	0.65	0.03	23.38	0.00	0.00
Total Right Cerebellar Cortex Volume[2]	Main Effect: Timepoint	0.65	0.03	23.23	0.00	0.00
Total Right Cerebellar Cortex Volume[3]	Main Effect: Timepoint	0.64	0.03	22.20	0.00	0.00
Total Right Cerebellar White Matter Volume[1]	Main Effect: Timepoint	0.38	0.02	21.98	0.00	0.00
Total Right Cerebellar White Matter Volume[2]	Main Effect: Timepoint	0.38	0.02	21.98	0.00	0.00
Total Right Cerebellar White Matter Volume[3]	Main Effect: Timepoint	0.39	0.02	21.60	0.00	0.00
Total Right Cortical White Matter Volume[1]	Main Effect: Timepoint	6.71	0.07	94.21	0.00	0.00

Total Right Cortical White Matter Volume[2]	Main Effect: Timepoint	6.71	0.07	93.76	0.00	0.00
Total Right Cortical White Matter Volume[3]	Main Effect: Timepoint	6.72	0.07	90.01	0.00	0.00
Total Subcortical Gray Matter Volume[1]	Main Effect: Timepoint	0.77	0.02	45.37	0.00	0.00
Total Subcortical Gray Matter Volume[2]	Main Effect: Timepoint	0.77	0.02	45.33	0.00	0.00
Total Subcortical Gray Matter Volume[3]	Main Effect: Timepoint	0.78	0.02	43.87	0.00	0.00

Supplementary Table 2 - *Group* by *Timepoint* interaction effects from multiple linear mixed effects regressions across 9 global brain volume measures representing moderated effects on developmental changes in brain volume between 10 to 12 years of age in the target group compared to the non-depressed control group. Regression models with sex and age as covaraites[1]; sex, age and self-reported race/ethnicity as covariates[2]; and sex, age, race/ethnicity, and SES indicators as covaraites[3].

sMRI Measure (outcome variable)	Regression Parameter (predictor variable)	Estimate (cm^3)	Std. Error	t value	p-value	p.adjusted	Significance
Total Brain Volume[1]	Interaction Effect: Depressed Controls by Timepoint	-5.19	2.28	-2.28	0.02	0.04	*
Total Brain Volume[2]	Interaction Effect: Depressed Controls by Timepoint	-5.21	2.28	-2.28	0.02	0.03	*
Total Brain Volume[3]	Interaction Effect: Depressed Controls by Timepoint	-6.30	2.39	-2.63	0.01	0.01	*
Total Cortical Gray Matter Volume[1]	Interaction Effect: Depressed Controls by Timepoint	-1.25	1.63	-0.77	0.44	0.49	
Total Cortical Gray Matter Volume[2]	Interaction Effect: Depressed Controls by Timepoint	-1.26	1.64	-0.77	0.44	0.48	
Total Cortical Gray Matter Volume[3]	Interaction Effect: Depressed Controls by Timepoint	-1.61	1.62	-1.00	0.32	0.36	
Total Left Cerebellar Cortex Volume[1]	Interaction Effect: Depressed Controls by Timepoint	-0.44	0.17	-2.60	0.01	0.02	*
Total Left Cerebellar Cortex Volume[2]	Interaction Effect: Depressed Controls by Timepoint	-0.44	0.17	-2.52	0.01	0.02	*
Total Left Cerebellar Cortex Volume[3]	Interaction Effect: Depressed Controls by Timepoint	-0.44	0.18	-2.41	0.02	0.02	*
Total Left Cerebellar White Matter Volume[1]	Interaction Effect: Depressed Controls by Timepoint	-0.17	0.14	-1.22	0.22	0.28	
Total Left Cerebellar White Matter Volume[2]	Interaction Effect: Depressed Controls by Timepoint	-0.16	0.13	-1.20	0.23	0.27	
Total Left Cerebellar White Matter Volume[3]	Interaction Effect: Depressed Controls by Timepoint	-0.23	0.14	-1.58	0.11	0.14	
Total Left Cortical White Matter Volume[1]	Interaction Effect: Depressed Controls by Timepoint	-1.30	0.51	-2.54	0.01	0.02	*
Total Left Cortical White Matter Volume[2]	Interaction Effect: Depressed Controls by Timepoint	-1.30	0.47	-2.76	0.01	0.01	*
Total Left Cortical White Matter Volume[3]	Interaction Effect: Depressed Controls by Timepoint	-1.66	0.56	-2.97	0.00	0.00	*
Total Right Cerebellar Cortex Volume[1]	Interaction Effect: Depressed Controls by Timepoint	0.07	0.19	0.37	0.71	0.74	
Total Right Cerebellar Cortex Volume[2]	Interaction Effect: Depressed Controls by Timepoint	0.07	0.20	0.35	0.72	0.74	
Total Right Cerebellar Cortex Volume[3]	Interaction Effect: Depressed Controls by Timepoint	0.08	0.20	0.37	0.71	0.74	
Total Right Cerebellar White Matter Volume[1]	Interaction Effect: Depressed Controls by Timepoint	-0.23	0.12	-1.87	0.06	0.09	
Total Right Cerebellar White Matter Volume[2]	Interaction Effect: Depressed Controls by Timepoint	-0.23	0.12	-1.87	0.06	0.08	
Total Right Cerebellar White Matter Volume[3]	Interaction Effect: Depressed Controls by Timepoint	-0.24	0.12	-1.95	0.05	0.07	
Total Right Cortical White Matter Volume[1]	Interaction Effect: Depressed Controls by Timepoint	-1.33	0.50	-2.64	0.01	0.01	*
Total Right Cortical White Matter Volume[2]	Interaction Effect: Depressed Controls by Timepoint	-1.33	0.49	-2.72	0.01	0.01	*
Total Right Cortical White Matter Volume[3]	Interaction Effect: Depressed Controls by Timepoint	-1.65	0.52	-3.14	0.00	0.00	*
Total Subcortical Gray Matter Volume[1]	Interaction Effect: Depressed Controls by Timepoint	-0.37	0.12	-2.97	0.00	0.01	*
Total Subcortical Gray Matter Volume[2]	Interaction Effect: Depressed Controls by Timepoint	-0.37	0.12	-2.97	0.00	0.00	*
Total Subcortical Gray Matter Volume[3]	Interaction Effect: Depressed Controls by Timepoint	-0.38	0.13	-2.95	0.00	0.00	*
Total Brain Volume[1]	Interaction Effect: Developed SB by Timepoint	-8.87	1.96	-4.52	0.00	0.00	*

Total Brain Volume[2]	Interaction Effect: Developed SB by Timepoint	-8.88	1.96	-4.53	0.00	0.00	*
Total Brain Volume[3]	Interaction Effect: Developed SB by Timepoint	-8.85	1.94	-4.56	0.00	0.00	*
Total Cortical Gray Matter Volume[1]	Interaction Effect: Developed SB by Timepoint	-6.01	1.40	-4.29	0.00	0.00	*
Total Cortical Gray Matter Volume[2]	Interaction Effect: Developed SB by Timepoint	-6.02	1.41	-4.27	0.00	0.00	*
Total Cortical Gray Matter Volume[3]	Interaction Effect: Developed SB by Timepoint	-6.03	1.49	-4.05	0.00	0.00	*
Total Left Cerebellar Cortex Volume[1]	Interaction Effect: Developed SB by Timepoint	-0.31	0.14	-2.13	0.03	0.05	
Total Left Cerebellar Cortex Volume[2]	Interaction Effect: Developed SB by Timepoint	-0.31	0.14	-2.13	0.03	0.05	*
Total Left Cerebellar Cortex Volume[3]	Interaction Effect: Developed SB by Timepoint	-0.32	0.14	-2.23	0.03	0.04	*
Total Left Cerebellar White Matter Volume[1]	Interaction Effect: Developed SB by Timepoint	-0.13	0.12	-1.09	0.27	0.33	
Total Left Cerebellar White Matter Volume[2]	Interaction Effect: Developed SB by Timepoint	-0.13	0.12	-1.10	0.27	0.31	
Total Left Cerebellar White Matter Volume[3]	Interaction Effect: Developed SB by Timepoint	-0.13	0.12	-1.06	0.29	0.33	
Total Left Cortical White Matter Volume[1]	Interaction Effect: Developed SB by Timepoint	-0.63	0.45	-1.42	0.16	0.21	
Total Left Cortical White Matter Volume[2]	Interaction Effect: Developed SB by Timepoint	-0.63	0.45	-1.41	0.16	0.19	
Total Left Cortical White Matter Volume[3]	Interaction Effect: Developed SB by Timepoint	-0.61	0.43	-1.40	0.16	0.19	
Total Right Cerebellar Cortex Volume[1]	Interaction Effect: Developed SB by Timepoint	-0.25	0.17	-1.53	0.13	0.17	
Total Right Cerebellar Cortex Volume[2]	Interaction Effect: Developed SB by Timepoint	-0.26	0.17	-1.51	0.13	0.17	
Total Right Cerebellar Cortex Volume[3]	Interaction Effect: Developed SB by Timepoint	-0.28	0.18	-1.61	0.11	0.14	
Total Right Cerebellar White Matter Volume[1]	Interaction Effect: Developed SB by Timepoint	-0.14	0.11	-1.31	0.19	0.24	
Total Right Cerebellar White Matter Volume[2]	Interaction Effect: Developed SB by Timepoint	-0.14	0.11	-1.28	0.20	0.24	
Total Right Cerebellar White Matter Volume[3]	Interaction Effect: Developed SB by Timepoint	-0.12	0.11	-1.17	0.24	0.28	
Total Right Cortical White Matter Volume[1]	Interaction Effect: Developed SB by Timepoint	-0.92	0.46	-2.02	0.04	0.07	
Total Right Cortical White Matter Volume[2]	Interaction Effect: Developed SB by Timepoint	-0.92	0.43	-2.14	0.03	0.05	*
Total Right Cortical White Matter Volume[3]	Interaction Effect: Developed SB by Timepoint	-0.88	0.46	-1.92	0.05	0.07	
Total Subcortical Gray Matter Volume[1]	Interaction Effect: Developed SB by Timepoint	-0.43	0.11	-4.12	0.00	0.00	*
Total Subcortical Gray Matter Volume[2]	Interaction Effect: Developed SB by Timepoint	-0.43	0.11	-4.12	0.00	0.00	*
Total Subcortical Gray Matter Volume[3]	Interaction Effect: Developed SB by Timepoint	-0.45	0.11	-4.12	0.00	0.00	*
Total Brain Volume[1]	Interaction Effect: Developed SI Only by Timepoint	-2.86	1.13	-2.54	0.01	0.02	*
Total Brain Volume[2]	Interaction Effect: Developed SI Only by Timepoint	-2.88	1.13	-2.55	0.01	0.02	*
Total Brain Volume[3]	Interaction Effect: Developed SI Only by Timepoint	-2.74	1.21	-2.27	0.02	0.03	*
Total Cortical Gray Matter Volume[1]	Interaction Effect: Developed SI Only by Timepoint	-2.13	0.85	-2.51	0.01	0.02	*
Total Cortical Gray Matter Volume[2]	Interaction Effect: Developed SI Only by Timepoint	-2.14	0.83	-2.59	0.01	0.01	*
Total Cortical Gray Matter Volume[3]	Interaction Effect: Developed SI Only by Timepoint	-2.18	0.85	-2.58	0.01	0.01	*
Total Left Cerebellar Cortex Volume[1]	Interaction Effect: Developed SI Only by Timepoint	-0.10	0.09	-1.13	0.26	0.31	
Total Left Cerebellar Cortex Volume[2]	Interaction Effect: Developed SI Only by Timepoint	-0.10	0.09	-1.15	0.25	0.29	
Total Left Cerebellar Cortex Volume[3]	Interaction Effect: Developed SI Only by Timepoint	-0.11	0.09	-1.22	0.22	0.26	

Total Left Cerebellar White Matter Volume[1]	Interaction Effect: Developed SI Only by Timepoint	-0.11	0.07	-1.56	0.12	0.16	
Total Left Cerebellar White Matter Volume[2]	Interaction Effect: Developed SI Only by Timepoint	-0.11	0.07	-1.58	0.11	0.15	
Total Left Cerebellar White Matter Volume[3]	Interaction Effect: Developed SI Only by Timepoint	-0.13	0.07	-1.85	0.06	0.08	
Total Left Cortical White Matter Volume[1]	Interaction Effect: Developed SI Only by Timepoint	-0.06	0.26	-0.23	0.82	0.85	
Total Left Cortical White Matter Volume[2]	Interaction Effect: Developed SI Only by Timepoint	-0.06	0.26	-0.24	0.81	0.83	
Total Left Cortical White Matter Volume[3]	Interaction Effect: Developed SI Only by Timepoint	0.04	0.26	0.17	0.87	0.89	
Total Right Cerebellar Cortex Volume[1]	Interaction Effect: Developed SI Only by Timepoint	-0.10	0.10	-0.98	0.33	0.38	
Total Right Cerebellar Cortex Volume[2]	Interaction Effect: Developed SI Only by Timepoint	-0.10	0.10	-1.01	0.31	0.36	
Total Right Cerebellar Cortex Volume[3]	Interaction Effect: Developed SI Only by Timepoint	-0.08	0.11	-0.74	0.46	0.50	
Total Right Cerebellar White Matter Volume[1]	Interaction Effect: Developed SI Only by Timepoint	-0.06	0.06	-0.94	0.35	0.40	
Total Right Cerebellar White Matter Volume[2]	Interaction Effect: Developed SI Only by Timepoint	-0.06	0.06	-0.97	0.33	0.38	
Total Right Cerebellar White Matter Volume[3]	Interaction Effect: Developed SI Only by Timepoint	-0.09	0.07	-1.29	0.20	0.23	
Total Right Cortical White Matter Volume[1]	Interaction Effect: Developed SI Only by Timepoint	-0.22	0.27	-0.82	0.41	0.46	
Total Right Cortical White Matter Volume[2]	Interaction Effect: Developed SI Only by Timepoint	-0.22	0.26	-0.85	0.40	0.44	
Total Right Cortical White Matter Volume[3]	Interaction Effect: Developed SI Only by Timepoint	-0.11	0.27	-0.42	0.68	0.71	
Total Subcortical Gray Matter Volume[1]	Interaction Effect: Developed SI Only by Timepoint	-0.11	0.06	-1.74	0.08	0.12	
Total Subcortical Gray Matter Volume[2]	Interaction Effect: Developed SI Only by Timepoint	-0.11	0.06	-1.75	0.08	0.11	
Total Subcortical Gray Matter Volume[3]	Interaction Effect: Developed SI Only by Timepoint	-0.10	0.07	-1.55	0.12	0.15	
Total Brain Volume[1]	Interaction Effect: Endorsed SB by Timepoint	-5.15	1.51	-3.42	0.00	0.00	*
Total Brain Volume[2]	Interaction Effect: Endorsed SB by Timepoint	-5.15	1.51	-3.42	0.00	0.00	*
Total Brain Volume[3]	Interaction Effect: Endorsed SB by Timepoint	-5.29	1.57	-3.37	0.00	0.00	*
Total Cortical Gray Matter Volume[1]	Interaction Effect: Endorsed SB by Timepoint	-3.09	1.07	-2.89	0.00	0.01	*
Total Cortical Gray Matter Volume[2]	Interaction Effect: Endorsed SB by Timepoint	-3.09	1.14	-2.72	0.01	0.01	*
Total Cortical Gray Matter Volume[3]	Interaction Effect: Endorsed SB by Timepoint	-3.23	1.11	-2.92	0.00	0.01	*
Total Left Cerebellar Cortex Volume[1]	Interaction Effect: Endorsed SB by Timepoint	-0.17	0.11	-1.48	0.14	0.18	
Total Left Cerebellar Cortex Volume[2]	Interaction Effect: Endorsed SB by Timepoint	-0.17	0.12	-1.42	0.15	0.19	
Total Left Cerebellar Cortex Volume[3]	Interaction Effect: Endorsed SB by Timepoint	-0.21	0.12	-1.74	0.08	0.11	
Total Left Cerebellar White Matter Volume[1]	Interaction Effect: Endorsed SB by Timepoint	-0.04	0.09	-0.47	0.64	0.68	
Total Left Cerebellar White Matter Volume[2]	Interaction Effect: Endorsed SB by Timepoint	-0.04	0.09	-0.47	0.64	0.67	
Total Left Cerebellar White Matter Volume[3]	Interaction Effect: Endorsed SB by Timepoint	-0.10	0.09	-1.11	0.27	0.31	
Total Left Cortical White Matter Volume[1]	Interaction Effect: Endorsed SB by Timepoint	-0.50	0.36	-1.39	0.17	0.21	
Total Left Cortical White Matter Volume[2]	Interaction Effect: Endorsed SB by Timepoint	-0.50	0.35	-1.45	0.15	0.18	
Total Left Cortical White Matter Volume[3]	Interaction Effect: Endorsed SB by Timepoint	-0.52	0.36	-1.42	0.15	0.19	
Total Right Cerebellar Cortex Volume[1]	Interaction Effect: Endorsed SB by Timepoint	-0.26	0.13	-1.92	0.05	0.09	
Total Right Cerebellar Cortex Volume[2]	Interaction Effect: Endorsed SB by Timepoint	-0.25	0.14	-1.88	0.06	0.08	

Total Right Cerebellar Cortex Volume[3]	Interaction Effect: Endorsed SB by Timepoint	-0.20	0.14	-1.44	0.15	0.18
Total Right Cerebellar White Matter Volume[1]	Interaction Effect: Endorsed SB by Timepoint	-0.07	0.08	-0.79	0.43	0.48
Total Right Cerebellar White Matter Volume[2]	Interaction Effect: Endorsed SB by Timepoint	-0.07	0.08	-0.78	0.44	0.48
Total Right Cerebellar White Matter Volume[3]	Interaction Effect: Endorsed SB by Timepoint	-0.08	0.08	-0.97	0.33	0.37
Total Right Cortical White Matter Volume[1]	Interaction Effect: Endorsed SB by Timepoint	-0.58	0.34	-1.70	0.09	0.13
Total Right Cortical White Matter Volume[2]	Interaction Effect: Endorsed SB by Timepoint	-0.58	0.34	-1.71	0.09	0.11
Total Right Cortical White Matter Volume[3]	Interaction Effect: Endorsed SB by Timepoint	-0.52	0.35	-1.47	0.14	0.17
Total Subcortical Gray Matter Volume[1]	Interaction Effect: Endorsed SB by Timepoint	-0.16	0.08	-1.86	0.06	0.09
Total Subcortical Gray Matter Volume[2]	Interaction Effect: Endorsed SB by Timepoint	-0.16	0.08	-1.86	0.06	0.09
Total Subcortical Gray Matter Volume[3]	Interaction Effect: Endorsed SB by Timepoint	-0.15	0.09	-1.75	0.08	0.10
Total Brain Volume[1]	Interaction Effect: Endorsed SI Only by Timepoint	-0.06	0.77	-0.08	0.94	0.94
Total Brain Volume[2]	Interaction Effect: Endorsed SI Only by Timepoint	-0.05	0.77	-0.06	0.95	0.95
Total Brain Volume[3]	Interaction Effect: Endorsed SI Only by Timepoint	-0.02	0.79	-0.02	0.98	0.98
Total Cortical Gray Matter Volume[1]	Interaction Effect: Endorsed SI Only by Timepoint	0.06	0.55	0.10	0.92	0.93
Total Cortical Gray Matter Volume[2]	Interaction Effect: Endorsed SI Only by Timepoint	0.07	0.56	0.13	0.90	0.91
Total Cortical Gray Matter Volume[3]	Interaction Effect: Endorsed SI Only by Timepoint	0.02	0.58	0.04	0.97	0.97
Total Left Cerebellar Cortex Volume[1]	Interaction Effect: Endorsed SI Only by Timepoint	-0.07	0.06	-1.17	0.24	0.29
Total Left Cerebellar Cortex Volume[2]	Interaction Effect: Endorsed SI Only by Timepoint	-0.07	0.06	-1.17	0.24	0.29
Total Left Cerebellar Cortex Volume[3]	Interaction Effect: Endorsed SI Only by Timepoint	-0.06	0.06	-0.95	0.34	0.38
Total Left Cerebellar White Matter Volume[1]	Interaction Effect: Endorsed SI Only by Timepoint	-0.04	0.05	-0.89	0.38	0.43
Total Left Cerebellar White Matter Volume[2]	Interaction Effect: Endorsed SI Only by Timepoint	-0.04	0.05	-0.87	0.39	0.43
Total Left Cerebellar White Matter Volume[3]	Interaction Effect: Endorsed SI Only by Timepoint	-0.05	0.05	-1.00	0.32	0.36
Total Left Cortical White Matter Volume[1]	Interaction Effect: Endorsed SI Only by Timepoint	-0.11	0.17	-0.62	0.53	0.57
Total Left Cortical White Matter Volume[2]	Interaction Effect: Endorsed SI Only by Timepoint	-0.11	0.17	-0.62	0.54	0.57
Total Left Cortical White Matter Volume[3]	Interaction Effect: Endorsed SI Only by Timepoint	-0.09	0.18	-0.47	0.64	0.68
Total Right Cerebellar Cortex Volume[1]	Interaction Effect: Endorsed SI Only by Timepoint	-0.05	0.07	-0.68	0.50	0.55
Total Right Cerebellar Cortex Volume[2]	Interaction Effect: Endorsed SI Only by Timepoint	-0.04	0.07	-0.66	0.51	0.55
Total Right Cerebellar Cortex Volume[3]	Interaction Effect: Endorsed SI Only by Timepoint	-0.03	0.07	-0.40	0.69	0.72
Total Right Cerebellar White Matter Volume[1]	Interaction Effect: Endorsed SI Only by Timepoint	-0.02	0.04	-0.58	0.56	0.60
Total Right Cerebellar White Matter Volume[2]	Interaction Effect: Endorsed SI Only by Timepoint	-0.02	0.04	-0.56	0.58	0.61
Total Right Cerebellar White Matter Volume[3]	Interaction Effect: Endorsed SI Only by Timepoint	-0.02	0.04	-0.42	0.68	0.71
Total Right Cortical White Matter Volume[1]	Interaction Effect: Endorsed SI Only by Timepoint	-0.11	0.17	-0.66	0.51	0.56
Total Right Cortical White Matter Volume[2]	Interaction Effect: Endorsed SI Only by Timepoint	-0.11	0.17	-0.65	0.52	0.55
Total Right Cortical White Matter Volume[3]	Interaction Effect: Endorsed SI Only by Timepoint	-0.12	0.18	-0.68	0.50	0.54
Total Subcortical Gray Matter Volume[1]	Interaction Effect: Endorsed SI Only by Timepoint	-0.05	0.04	-1.21	0.22	0.28

Total Subcortical Gray Matter Volume[2]	Interaction Effect: Endorsed SI Only by Timepoint	-0.05	0.04	-1.20	0.23	0.27
Total Subcortical Gray Matter Volume[3]	Interaction Effect: Endorsed SI Only by Timepoint	-0.07	0.04	-1.60	0.11	0.14

Supplementary Table 3 - Comparison of *Group* by *Timepoint* interaction effects representing differences in the rates of developmental change in brain volume between specified groups. These comparisons were assessed using general linear hypotheses post-hoc contrasts which tests the null hypothesis of no significant difference between two specified *group* by *timepoint* interaction effects (ex. Developed SB by Timepoint - Endorsed SB by Timepoint = 0).

Brain Measure	Contrasts	Estimate (cm3)	Standard Error	Z-value	P-value (adjusted)	Significance
Total Brain Volume	Developed SB by Timepoint - Depressed Controls by Timepoint = 0	-2.544	2.516	-1.011	0.464	
Total Brain Volume	Developed SB by Timepoint - Developed SI Only by Timepoint = 0	-6.109	1.883	-3.245	0.015	*
Total Brain Volume	Developed SB by Timepoint - Endorsed SB by Timepoint = 0	-3.559	2.063	-1.725	0.220	
Total Cortical Gray Matter Volume Volume	Developed SB by Timepoint - Developed SI Only by Timepoint = 0	-3.846	1.370	-2.807	0.030	*
Total Cortical Gray Matter Volume Volume	Developed SB by Timepoint - Endorsed SB by Timepoint = 0	-2.794	1.504	-1.858	0.137	
Cerebellar Cortex Left	Developed SB by Timepoint - Depressed Controls by Timepoint = 0	0.118	0.193	0.611	0.865	
Sub-Cortical Gray Volume	Developed SB by Timepoint - Depressed Controls by Timepoint = 0	-0.066	0.136	-0.484	0.707	

Supplementary Table 4 - Assessment of estimated differences in brain volume measures between those who developed SB and other groups at each timepoint using general linear hypotheses post-hoc contrasts which tests the null hypothesis of no significant difference in brain volume between two specified groups at a specified timepoint (ex. Developed SB - Endorsed SI at Baseline = 0). Note, contrasts between those who developed SB and other non-depressed controls at baseline were already estimated as main effects in the original regression models and were not included here.

Brain Measure	Contrasts	Estimate	Standard Error	Z-value	P-value (adjusted)	Significance
Total Brain Volume	Developed SB - Developed SI Only at Baseline = 0	-12.062	8.792	-1.372	0.369	
Total Brain Volume	Developed SB - Endorsed SB at Baseline = 0	1.352	9.719	0.139	0.889	
Total Brain Volume	Developed SB - Endorsed SI at Baseline = 0	-8.698	8.154	-1.067	0.464	
Total Brain Volume	Developed SB - Depressed Controls at Baseline = 0	6.736	11.394	0.591	0.721	
Total Brain Volume	Developed SB - Depressed SI Only at Two-Year Follow-Up = 0	-18.171	8.843	-2.055	0.130	
Total Brain Volume	Developed SB - Endorsed SB at Two-Year Follow-Up = 0	-2.207	9.768	-0.226	0.889	
Total Brain Volume	Developed SB - Endorsed SI Only at Two-Year Follow-Up = 0	-17.530	8.199	-2.138	0.130	
Total Brain Volume	Developed SB - Depressed Controls at Two-Year Follow-Up = 0	4.192	11.482	0.365	0.845	
Total Brain Volume	Developed SB - Non Depressed Controls at Two-Year Follow-Up = 0	-21.023	7.770	-2.706	0.044	*
Total Cortical Gray Matter Volume Volume	Developed SB - Developed SI Only at Baseline = 0	-4.236	4.382	-0.967	0.501	
Total Cortical Gray Matter Volume Volume	Developed SB - Endorsed SB at Baseline = 0	2.316	4.835	0.479	0.689	
Total Cortical Gray Matter Volume Volume	Developed SB - Endorsed SI at Baseline = 0	-2.994	4.062	-0.737	0.615	

Total Cortical Gray Matter Volume Volume	Developed SB - Depressed Controls at Baseline = 0	7.932	5.650	1.404	0.275	
Total Cortical Gray Matter Volume Volume	Developed SB - Depressed SI Only at Two-Year Follow-Up = 0	-8.083	4.438	-1.821	0.137	
Total Cortical Gray Matter Volume Volume	Developed SB - Endorsed SB at Two-Year Follow-Up = 0	-0.478	4.890	-0.098	0.922	
Total Cortical Gray Matter Volume Volume	Developed SB - Endorsed SI Only at Two-Year Follow-Up = 0	-9.046	4.112	-2.200	0.083	
Total Cortical Gray Matter Volume Volume	Developed SB - Depressed Controls at Two-Year Follow-Up = 0	3.515	5.743	0.612	0.649	
Total Cortical Gray Matter Volume Volume	Developed SB - Non Depressed Controls at Two-Year Follow-Up = 0	-10.436	3.896	-2.679	0.030	*
Cerebral White Matter Left	Developed SB - Developed SI Only at Baseline = 0	-3.302	1.992	-1.658	0.195	
Cerebral White Matter Left	Developed SB - Endorsed SB at Baseline = 0	-1.092	2.201	-0.496	0.754	
Cerebral White Matter Left	Developed SB - Endorsed SI at Baseline = 0	-2.707	1.848	-1.465	0.238	
Cerebral White Matter Left	Developed SB - Depressed Controls at Baseline = 0	0.026	2.582	0.010	0.992	
Cerebral White Matter Left	Developed SB - Depressed SI Only at Two-Year Follow-Up = 0	-3.953	2.003	-1.973	0.162	
Cerebral White Matter Left	Developed SB - Endorsed SB at Two-Year Follow-Up = 0	-1.181	2.212	-0.534	0.754	
Cerebral White Matter Left	Developed SB - Endorsed SI Only at Two-Year Follow-Up = 0	-3.229	1.858	-1.738	0.195	
Cerebral White Matter Left	Developed SB - Depressed Controls at Two-Year Follow-Up = 0	1.079	2.601	0.415	0.754	
Cerebral White Matter Left	Developed SB - Non Depressed Controls at Two-Year Follow-Up = 0	-3.777	1.760	-2.146	0.159	
Cerebral White Matter Right	Developed SB - Developed SI Only at Baseline = 0	-2.991	1.989	-1.504	0.265	

Cerebral White Matter Right	Developed SB - Endorsed SB at Baseline = 0	-0.875	2.198	-0.398	0.791
Cerebral White Matter Right	Developed SB - Endorsed SI at Baseline = 0	-2.487	1.845	-1.348	0.296
Cerebral White Matter Right	Developed SB - Depressed Controls at Baseline = 0	0.191	2.578	0.074	0.941
Cerebral White Matter Right	Developed SB - Depressed SI Only at Two-Year Follow-Up = 0	-3.755	2.001	-1.877	0.201
Cerebral White Matter Right	Developed SB - Endorsed SB at Two-Year Follow-Up = 0	-1.230	2.209	-0.557	0.791
Cerebral White Matter Right	Developed SB - Endorsed SI Only at Two-Year Follow-Up = 0	-3.245	1.855	-1.749	0.201
Cerebral White Matter Right	Developed SB - Depressed Controls at Two-Year Follow-Up = 0	0.959	2.598	0.369	0.791
Cerebral White Matter Right	Developed SB - Non Depressed Controls at Two-Year Follow-Up = 0	-3.902	1.757	-2.220	0.132
Cerebellar Cortex Left	Developed SB - Developed SI Only at Baseline = 0	-0.027	0.463	-0.059	0.987
Cerebellar Cortex Left	Developed SB - Endorsed SB at Baseline = 0	0.361	0.511	0.706	0.865
Cerebellar Cortex Left	Developed SB - Endorsed SI at Baseline = 0	-0.208	0.429	-0.483	0.865
Cerebellar Cortex Left	Developed SB - Depressed Controls at Baseline = 0	0.010	0.601	0.017	0.987
Cerebellar Cortex Left	Developed SB - Depressed SI Only at Two-Year Follow-Up = 0	-0.240	0.469	-0.512	0.865
Cerebellar Cortex Left	Developed SB - Endorsed SB at Two-Year Follow-Up = 0	0.250	0.517	0.484	0.865
Cerebellar Cortex Left	Developed SB - Endorsed SI Only at Two-Year Follow-Up = 0	-0.472	0.434	-1.087	0.865
Cerebellar Cortex Left	Developed SB - Depressed Controls at Two-Year Follow-Up = 0	0.128	0.611	0.210	0.987

Cerebellar Cortex Left	Developed SB - Non Depressed Controls at Two-Year Follow-Up = 0	-0.540	0.411	-1.314	0.865
Cerebellar Cortex right	Developed SB - Developed SI Only at Baseline = 0	-0.127	0.477	-0.265	0.939
Cerebellar Cortex right	Developed SB - Endorsed SB at Baseline = 0	0.192	0.528	0.365	0.939
Cerebellar Cortex right	Developed SB - Endorsed SI at Baseline = 0	-0.215	0.443	-0.487	0.939
Cerebellar Cortex right	Developed SB - Depressed Controls at Baseline = 0	0.040	0.621	0.064	0.949
Cerebellar Cortex right	Developed SB - Depressed SI Only at Two-Year Follow-Up = 0	-0.331	0.485	-0.682	0.939
Cerebellar Cortex right	Developed SB - Endorsed SB at Two-Year Follow-Up = 0	0.112	0.535	0.209	0.939
Cerebellar Cortex right	Developed SB - Endorsed SI Only at Two-Year Follow-Up = 0	-0.470	0.449	-1.046	0.939
Cerebellar Cortex right	Developed SB - Depressed Controls at Two-Year Follow-Up = 0	-0.319	0.634	-0.503	0.939
Cerebellar Cortex right	Developed SB - Non Depressed Controls at Two-Year Follow-Up = 0	-0.616	0.425	-1.449	0.939
Cerebellar White Matter Left	Developed SB - Developed SI Only at Baseline = 0	-0.112	0.165	-0.680	0.978
Cerebellar White Matter Left	Developed SB - Endorsed SB at Baseline = 0	0.005	0.183	0.028	0.978
Cerebellar White Matter Left	Developed SB - Endorsed SI at Baseline = 0	-0.016	0.153	-0.105	0.978
Cerebellar White Matter Left	Developed SB - Depressed Controls at Baseline = 0	-0.050	0.214	-0.232	0.978
Cerebellar White Matter Left	Developed SB - Depressed SI Only at Two-Year Follow-Up = 0	-0.111	0.175	-0.636	0.978
Cerebellar White Matter Left	Developed SB - Endorsed SB at Two-Year Follow-Up = 0	-0.023	0.192	-0.119	0.978

Cerebellar White Matter Left	Developed SB - Endorsed SI Only at Two-Year Follow-Up = 0	-0.099	0.162	-0.613	0.978	
Cerebellar White Matter Left	Developed SB - Depressed Controls at Two-Year Follow-Up = 0	0.045	0.231	0.195	0.978	
Cerebellar White Matter Left	Developed SB - Non Depressed Controls at Two-Year Follow-Up = 0	-0.187	0.153	-1.223	0.978	
Cerebellar White Matter right	Developed SB - Developed SI Only at Baseline = 0	-0.109	0.152	-0.714	0.951	
Cerebellar White Matter right	Developed SB - Endorsed SB at Baseline = 0	-0.059	0.169	-0.351	0.999	
Cerebellar White Matter right	Developed SB - Endorsed SI at Baseline = 0	-0.099	0.142	-0.697	0.956	
Cerebellar White Matter right	Developed SB - Depressed Controls at Baseline = 0	-0.141	0.196	-0.718	0.950	
Cerebellar White Matter right	Developed SB - Depressed SI Only at Two-Year Follow-Up = 0	-0.149	0.161	-0.926	0.855	
Cerebellar White Matter right	Developed SB - Endorsed SB at Two-Year Follow-Up = 0	-0.102	0.177	-0.577	0.983	
Cerebellar White Matter right	Developed SB - Endorsed SI Only at Two-Year Follow-Up = 0	-0.205	0.149	-1.381	0.541	
Cerebellar White Matter right	Developed SB - Depressed Controls at Two-Year Follow-Up = 0	-0.026	0.211	-0.123	1.000	
Cerebellar White Matter right	Developed SB - Non Depressed Controls at Two-Year Follow-Up = 0	-0.282	0.141	-2.005	0.192	
Sub-Cortical Gray Volume	Developed SB - Endorsed SB at Baseline = 0	-0.075	0.445	-0.169	0.865	
Sub-Cortical Gray Volume	Developed SB - Endorsed SI at Baseline = 0	-0.353	0.373	-0.948	0.707	
Sub-Cortical Gray Volume	Developed SB - Developed SI Only at Baseline = 0	-0.606	0.402	-1.506	0.291	
Sub-Cortical Gray Volume	Developed SB - Depressed Controls at Baseline = 0	-0.265	0.521	-0.508	0.707	

Sub-Cortical Gray Volume	Developed SB - Endorsed SB at Two-Year Follow-Up = 0	-0.373	0.449	-0.831	0.707	
Sub-Cortical Gray Volume	Developed SB - Endorsed SI Only at Two-Year Follow-Up = 0	-0.734	0.376	-1.952	0.153	
Sub-Cortical Gray Volume	Developed SB - Developed SI Only at Two-Year Follow-Up = 0	-0.954	0.406	-2.351	0.069	
Sub-Cortical Gray Volume	Developed SB - Depressed Controls at Two-Year Follow-Up = 0	-0.331	0.527	-0.628	0.707	
Sub-Cortical Gray Volume	Developed SB - Non Depressed Controls at Two-Year Follow-Up = 0	-1.003	0.356	-2.816	0.022	*

Supplementary Table 5 - Sensitivity Analysis For Total Brain Volume Regression. A sensitivity analysis was carried out controlling for birth weight (adjusted for th enumber of weeks premature), and alcohol use.

Regression Parameters	Original Analysis Regression Estimates	Sensitivity Analysis Regression Estimates
**STB Group**		
Non-Depressed Controls	—	—
Depressed Controls	-19 (-36 to -2.2)	-16 (-34 to 0.69)
Endorsed SI Only	-3.5 (-9.4 to 2.5)	-2.3 (-8.3 to 3.7)
Developed SI Only	-0.11 (-8.9 to 8.7)	0.68 (-8.1 to 9.4)
Endorsed SB	-14 (-26 to -1.5)	-10 (-22 to 1.9)
Developed SB	-12 (-27 to 3.0)	-8.4 (-24 to 7.4)
**Timepoint**		
Baseline	—	—
Two-year follow-up	7.2 (6.7 to 7.8)	7.2 (6.7 to 7.8)
**Sex**		
Male	—	—
Female	-108 (-112 to -104)	-104 (-108 to -100)
**Age (baseline)**	0.04 (-0.19 to 0.27)	0.18 (-0.06 to 0.41)
**Race/Ethnicity**		
white	—	—
black	-75 (-82 to -68)	-68 (-75 to -61)
hispanic	-36 (-43 to -30)	-33 (-39 to -27)
asian	-51 (-67 to -34)	-41 (-59 to -24)
other	-26 (-33 to -19)	-25 (-32 to -18)
**Poverty Indicator**		
No Poverty Indicator	—	—
One Poverty Indicator	-6.4 (-14 to 0.94)	-7.9 (-15 to -0.46)
Two or More Poverty Indicators	-8.1 (-15 to -1.5)	-8.4 (-15 to -1.5)
**Combined Family Income**		

Less thank 50K	—	—
Between 50K and 100K	7.5 (1.4 to 14)	5.9 (-0.30 to 12)
Above 100K	16 (9.6 to 23)	14 (7.0 to 21)
**Highest Parental Education**		
High School or Less	—	—
Post-Secondary but No College	6.7 (-1.0 to 14)	8.7 (0.60 to 17)
Undergraduate or Above	19 (11 to 27)	20 (11 to 28)
**Total Birth Weight (oz)**	NA	22 (19 to 24)
**Number of Weeks Premature**	NA	8.1 (5.6 to 11)
**Ever Sipped or Drank a Full Drink of Alcohol**		
no	NA	—
yes	NA	-0.14 (-4.4 to 4.1)
**STB Group by Timepoint**		
Depressed Controls by Two-Year Follow-Up	-6.3 (-10 to -2.5)	-6.3 (-10 to -2.2)
Endorsed SI Only by Two-Year Follow-Up	-0.02 (-1.3 to 1.3)	-0.07 (-1.4 to 1.2)
Developed SI Only by Two-Year Follow-Up	-2.7 (-4.7 to -0.80)	-2.7 (-4.7 to -0.77)
Endorsed SB by Two-Year Follow-Up	-5.3 (-7.8 to -2.8)	-5.0 (-7.6 to -2.4)
Developed SB by Two-Year Follow-Up	-8.8 (-12 to -5.6)	-7.6 (-11 to -4.3)
**Sex by Timepoint**		
Female by Two-Year Follow-Up	-3.5 (-4.3 to -2.8)	-3.3 (-4.1 to -2.5)
**Random Effects**		
site:rel_family_id:subjectkey.sd (Intercept)	57	55
site:rel_family_id.sd (Intercept)	69	69
site.sd (Intercept)	11	11
Residual.sd	11	11

Supplementary Table 6 - Total estimated change in brain volume measures between 10 to 12 years of age for a specified group assessed using general linear hypotheses post-hoc contrasts which tests the null hypothesis of no significant total effect of *timepoint* and *group by timepoint* interaction effects combined (i.e. Timepoint + Developed SB by Timepoint = 0).

Brain Measure	Contrasts	Estimate	Standard Error	Z-value	P-value (adjusted)	Significance
Total Brain Volume	Timepoint + Developed SB by Timepoint = 0	-1.623	1.636	-0.992	0.464	
Total Cortical Gray Matter Volume Volume	Timepoint + Developed SB by Timepoint = 0	-15.215	1.192	-12.759	0.000	*
Cerebral White Matter Left	Timepoint + Depressed Controls by Timepoint = 0	5.080	0.431	11.790	0.000	*
Cerebral White Matter Right	Timepoint + Depressed Controls by Timepoint = 0	5.074	0.433	11.720	0.000	*
Cerebellar Cortex Left	Timepoint + Developed SB by Timepoint = 0	0.087	0.149	0.586	0.865	
Sub-Cortical Gray Volume	Timepoint + Developed SB by Timepoint = 0	0.327	0.089	3.686	0.002	*

Supplementary Table 7 - Sensitivity Analysis For Total Cortical Gray Matter Volume Regression. A sensitivity analysis was carried out controlling for birth weight (adjusted for the number of weeks premature), and alcohol use.

Regression Parameters	Original Analysis Regression Estimates (95% CI)	Sensitivity Analysis Regression Estimates (95% CI)
**STB Group**		
Non-Depressed Controls	—	—
Depressed Controls	-12 (-21 to -4.1)	-11 (-20 to -2.9)
Endorsed SI Only	-1.4 (-4.4 to 1.6)	-0.85 (-3.9 to 2.2)
Developed SI Only	-0.17 (-4.6 to 4.2)	0.22 (-4.2 to 4.6)
Endorsed SB	-6.7 (-13 to -0.77)	-4.9 (-11 to 1.1)
Developed SB	-4.4 (-12 to 3.1)	-2.3 (-10 to 5.5)
**Timepoint**		
Baseline	—	—
Two-year follow-up	-9.2 (-9.6 to -8.8)	-9.2 (-9.6 to -8.8)
**Sex**		
Male	—	—
Female	-48 (-50 to -46)	-46 (-48 to -44)
**Age (baseline)**	-0.42 (-0.53 to -0.30)	-0.35 (-0.47 to -0.24)
**Race/Ethnicity**		
white	—	—
black	-42 (-46 to -39)	-39 (-43 to -35)
hispanic	-19 (-22 to -16)	-17 (-20 to -14)
asian	-27 (-35 to -19)	-23 (-31 to -14)
other	-16 (-19 to -13)	-15 (-19 to -12)
**Poverty Indicator**		
No Poverty Indicator	—	—
One Poverty Indicator	-3.2 (-6.8 to 0.38)	-3.9 (-7.6 to -0.22)

Two or More Poverty Indicators	-4.7 (-8.0 to -1.4)	-4.7 (-8.1 to -1.3)
**Combined Family Income**		
Less thank 50K	—	—
Between 50K and 100K	3.6 (0.62 to 6.6)	2.8 (-0.27 to 5.8)
Above 100K	8.3 (4.9 to 12)	7.0 (3.6 to 10)
**Highest Parental Education**		
High School or Less	—	—
Post-Secondary but No College	3.3 (-0.49 to 7.2)	4.4 (0.38 to 8.4)
Undergraduate or Above	10 (6.3 to 15)	11 (6.8 to 15)
**Total Birth Weight (oz)**	NA	10 (9.1 to 12)
**Number of Weeks Premature**	NA	3.1 (1.9 to 4.3)
**Ever Sipped or Drank a Full Drink of Alcohol**		
no	NA	—
yes	NA	0.58 (-1.5 to 2.7)
**STB Group by Timepoint**		
Depressed Controls by Two-Year Follow-Up	-1.6 (-4.3 to 1.1)	-1.2 (-4.1 to 1.7)
Endorsed SI Only by Two-Year Follow-Up	0.02 (-0.90 to 0.95)	0.04 (-0.90 to 0.99)
Developed SI Only by Two-Year Follow-Up	-2.2 (-3.6 to -0.78)	-2.2 (-3.6 to -0.78)
Endorsed SB by Two-Year Follow-Up	-3.2 (-5.1 to -1.4)	-3.2 (-5.1 to -1.3)
Developed SB by Two-Year Follow-Up	-6.0 (-8.4 to -3.7)	-5.3 (-7.7 to -2.8)
**Sex by Timepoint**		
Female by Two-Year Follow-Up	-2.8 (-3.3 to -2.2)	-2.6 (-3.2 to -2.1)
**Random Effects**		
site:rel_family_id:subjectkey.sd (Intercept)	28 (NA to NA)	27 (NA to NA)
site:rel_family_id.sd (Intercept)	34 (NA to NA)	34 (NA to NA)
site.sd (Intercept)	6.8 (NA to NA)	6.1 (NA to NA)
Residual.sd	7.8 (NA to NA)	7.8 (NA to NA)

Supplementary Table 8 - Main effect of *Timepoint* from multiple linear mixed effects regressions for 32 behavioral and psychiatric measures representing changes in these measures in the non-depressed control group between 10 and 12 years of age. Regression models included sex, age, race/ethnicity, and SES indicators as covariates. Positive estimates indicate increases in and a negative estimates indicate decreases in the respective behavioral and psychiatric measures.

Psychiatric/Behavioral Measures	Regression Parameters	Estimate	Std. Error	t value	p-value	p.adjusted
BIS/BAS: Drive	Main Effect: Timepoint	-0.130	0.018	-7.067	0.000	0.000
BIS/BAS: Fun Seeking	Main Effect: Timepoint	-0.404	0.019	-21.626	0.000	0.000
BIS/BAS: Reward Responsiveness	Main Effect: Timepoint	-0.370	0.019	-19.783	0.000	0.000
BIS/BAS: Drive Modified	Main Effect: Timepoint	-0.130	0.018	-7.067	0.000	0.000
BIS/BAS: Reward Responsiveness Modified	Main Effect: Timepoint	-0.363	0.019	-19.239	0.000	0.000
BIS/BAS: BIS Sum Score	Main Effect: Timepoint	-0.271	0.018	-14.850	0.000	0.000
BIS/BAS: BIS Sum Score Modified	Main Effect: Timepoint	-0.264	0.019	-14.276	0.000	0.000
CBCL: OCD	Main Effect: Timepoint	-0.042	0.007	-6.119	0.000	0.000
CBCL: Sluggish Cognitive Tempo	Main Effect: Timepoint	-0.009	0.005	-1.619	0.106	0.160
CBCL: Stress	Main Effect: Timepoint	-0.039	0.007	-5.349	0.000	0.000
CBCL: DSM5 ADHD	Main Effect: Timepoint	-0.053	0.008	-6.947	0.000	0.000
CBCL: DSM5 Anxiety Disorder	Main Effect: Timepoint	-0.070	0.008	-9.181	0.000	0.000
CBCL: DSM5 Conduct	Main Effect: Timepoint	-0.024	0.005	-5.341	0.000	0.000
CBCL: DSM5 Depression	Main Effect: Timepoint	0.020	0.006	3.439	0.001	0.001
CBCL: DSM5 Oppositional Defiance	Main Effect: Timepoint	-0.043	0.008	-5.287	0.000	0.000
CBCL: DSM5 Somatic Problems	Main Effect: Timepoint	-0.007	0.007	-1.001	0.317	0.404
CBCL: Aggressive Syndrome	Main Effect: Timepoint	-0.036	0.006	-6.466	0.000	0.000
CBCL: Anxious Depression Syndrome	Main Effect: Timepoint	-0.053	0.007	-7.457	0.000	0.000
CBCL: Attention Problems	Main Effect: Timepoint	-0.036	0.007	-5.090	0.000	0.000
CBCL: Externalizing	Main Effect: Timepoint	-0.038	0.005	-7.141	0.000	0.000
CBCL: Internalizing	Main Effect: Timepoint	-0.038	0.008	-4.943	0.000	0.000
CBCL: Rule-Breaking	Main Effect: Timepoint	-0.033	0.005	-5.963	0.000	0.000
CBCL: Social Problems	Main Effect: Timepoint	-0.064	0.006	-10.875	0.000	0.000
CBCL: Somatic Syndrome	Main Effect: Timepoint	-0.020	0.007	-2.791	0.005	0.010
CBCL: Thought Problems	Main Effect: Timepoint	-0.052	0.006	-8.258	0.000	0.000

CBCL: Total Problems	Main Effect: Timepoint	-0.082	0.009	-8.979	0.000	0.000
CBCL: Depression Syndrome	Main Effect: Timepoint	0.017	0.006	2.970	0.003	0.006
UPPS: Lack of Perserverance	Main Effect: Timepoint	-0.086	0.018	-4.830	0.000	0.000
UPPS: Lack of Planning	Main Effect: Timepoint	-0.018	0.018	-1.018	0.309	0.395
UPPS: Negative Urgency	Main Effect: Timepoint	-0.309	0.019	-16.503	0.000	0.000
UPPS: Positive Urgency	Main Effect: Timepoint	-0.193	0.019	-10.410	0.000	0.000
UPPS: Sensation Seeking	Main Effect: Timepoint	-0.107	0.017	-6.182	0.000	0.000

Supplementary Table 9 - *Group* by *Timepoint* interaction effects from multiple linear mixed effects regressions across 32 behavioral and psychiatric measures representing moderated effects on developmental changes in these measures between 10 to 12 years of age in the target group compared to the non-depressed control group. Regression models included sex, age, race/ethnicity, and SES indicators as covaraites. Bolded rows indicate significant *group* by *timepoint* interaction effects for those who developed SB.

Psychiatric/Behavioral Measures	Regression Parameters	Estimate	Std. Error	t value	Pr(>|t|)	p.adjusted	Significance
BIS/BAS: BIS Sum Score	Interaction Effect: Depressed Controls by Timepoint	0.232	0.125	1.856	0.063	0.102	
BIS/BAS: BIS Sum Score	Interaction Effect: Developed SI Only by Timepoint	0.309	0.064	4.796	0.000	0.000	*
BIS/BAS: BIS Sum Score	Interaction Effect: Endorsed SB by Timepoint	-0.132	0.086	-1.523	0.128	0.190	
BIS/BAS: BIS Sum Score	Interaction Effect: Endorsed SI Only by Timepoint	-0.001	0.044	-0.012	0.990	0.991	
BIS/BAS: BIS Sum Score	Interaction Effect: Developed SB by Timepoint	0.212	0.109	1.949	0.051	0.085	
BIS/BAS: BIS Sum Score Modified	Interaction Effect: Depressed Controls by Timepoint	0.179	0.127	1.410	0.158	0.227	
BIS/BAS: BIS Sum Score Modified	Interaction Effect: Developed SI Only by Timepoint	0.281	0.065	4.301	0.000	0.000	*
BIS/BAS: BIS Sum Score Modified	Interaction Effect: Endorsed SB by Timepoint	-0.151	0.088	-1.719	0.086	0.133	
BIS/BAS: BIS Sum Score Modified	Interaction Effect: Endorsed SI Only by Timepoint	-0.057	0.045	-1.278	0.201	0.277	
BIS/BAS: BIS Sum Score Modified	Interaction Effect: Developed SB by Timepoint	0.209	0.110	1.894	0.058	0.095	
BIS/BAS: Drive	Interaction Effect: Depressed Controls by Timepoint	-0.018	0.126	-0.145	0.885	0.914	
BIS/BAS: Drive	Interaction Effect: Developed SI Only by Timepoint	-0.008	0.065	-0.119	0.905	0.929	

BIS/BAS: Drive	Interaction Effect: Endorsed SB by Timepoint	-0.189	0.087	-2.169	0.030	0.052	
BIS/BAS: Drive	Interaction Effect: Endorsed SI Only by Timepoint	-0.165	0.044	-3.717	0.000	0.000	*
BIS/BAS: Drive	Interaction Effect: Developed SB by Timepoint	0.120	0.110	1.093	0.274	0.359	
BIS/BAS: Drive Modified	Interaction Effect: Depressed Controls by Timepoint	-0.018	0.126	-0.145	0.885	0.914	
BIS/BAS: Drive Modified	Interaction Effect: Developed SI Only by Timepoint	-0.008	0.065	-0.119	0.905	0.929	
BIS/BAS: Drive Modified	Interaction Effect: Endorsed SB by Timepoint	-0.189	0.087	-2.169	0.030	0.052	
BIS/BAS: Drive Modified	Interaction Effect: Endorsed SI Only by Timepoint	-0.165	0.044	-3.717	0.000	0.000	*
BIS/BAS: Drive Modified	Interaction Effect: Developed SB by Timepoint	0.120	0.110	1.093	0.274	0.359	
BIS/BAS: Fun Seeking	Interaction Effect: Depressed Controls by Timepoint	0.095	0.128	0.747	0.455	0.539	
BIS/BAS: Fun Seeking	Interaction Effect: Developed SI Only by Timepoint	0.095	0.066	1.437	0.151	0.218	
BIS/BAS: Fun Seeking	Interaction Effect: Endorsed SB by Timepoint	-0.272	0.088	-3.077	0.002	0.004	*
BIS/BAS: Fun Seeking	Interaction Effect: Endorsed SI Only by Timepoint	-0.106	0.045	-2.363	0.018	0.033	*
**BIS/BAS: Fun Seeking**	**Interaction Effect: Developed SB by Timepoint**	**0.252**	**0.111**	**2.269**	**0.023**	**0.041**	*
BIS/BAS: Reward Responsiveness	Interaction Effect: Depressed Controls by Timepoint	-0.024	0.128	-0.190	0.849	0.887	
BIS/BAS: Reward Responsiveness	Interaction Effect: Developed SI Only by Timepoint	0.154	0.066	2.336	0.020	0.035	*
BIS/BAS: Reward Responsiveness	Interaction Effect: Endorsed SB by Timepoint	-0.146	0.088	-1.650	0.099	0.151	

BIS/BAS: Reward Responsiveness	Interaction Effect: Endorsed SI Only by Timepoint	-0.040	0.045	-0.882	0.378	0.464	
BIS/BAS: Reward Responsiveness	Interaction Effect: Developed SB by Timepoint	-0.002	0.111	-0.017	0.987	0.989	
BIS/BAS: Reward Responsiveness Modified	Interaction Effect: Depressed Controls by Timepoint	-0.007	0.129	-0.052	0.958	0.966	
BIS/BAS: Reward Responsiveness Modified	Interaction Effect: Developed SI Only by Timepoint	0.157	0.066	2.361	0.018	0.033	*
BIS/BAS: Reward Responsiveness Modified	Interaction Effect: Endorsed SB by Timepoint	-0.164	0.089	-1.845	0.065	0.104	
BIS/BAS: Reward Responsiveness Modified	Interaction Effect: Endorsed SI Only by Timepoint	-0.036	0.045	-0.798	0.425	0.510	
BIS/BAS: Reward Responsiveness Modified	Interaction Effect: Developed SB by Timepoint	-0.010	0.112	-0.085	0.932	0.947	
CBCL: Aggressive Syndrome	Interaction Effect: Depressed Controls by Timepoint	-0.016	0.038	-0.412	0.680	0.753	
CBCL: Aggressive Syndrome	Interaction Effect: Developed SB by Timepoint	0.049	0.035	1.395	0.163	0.232	
CBCL: Aggressive Syndrome	Interaction Effect: Developed SI Only by Timepoint	0.018	0.020	0.903	0.367	0.453	
CBCL: Aggressive Syndrome	Interaction Effect: Endorsed SB by Timepoint	-0.011	0.026	-0.424	0.672	0.746	
CBCL: Aggressive Syndrome	Interaction Effect: Endorsed SI Only by Timepoint	-0.009	0.013	-0.692	0.489	0.574	
**CBCL: Anxious Depression Syndrome**	**Interaction Effect: Developed SB by Timepoint**	**0.101**	**0.044**	**2.286**	**0.022**	**0.039**	*

CBCL: Anxious Depression Syndrome	Interaction Effect: Depressed Controls by Timepoint	-0.008	0.048	-0.170	0.865	0.900	
CBCL: Anxious Depression Syndrome	Interaction Effect: Developed SI Only by Timepoint	0.059	0.025	2.326	0.020	0.036	*
CBCL: Anxious Depression Syndrome	Interaction Effect: Endorsed SB by Timepoint	-0.040	0.033	-1.199	0.230	0.311	
CBCL: Anxious Depression Syndrome	Interaction Effect: Endorsed SI Only by Timepoint	-0.016	0.017	-0.972	0.331	0.420	
CBCL: Attention Problems	Interaction Effect: Depressed Controls by Timepoint	0.010	0.048	0.212	0.832	0.876	
CBCL: Attention Problems	Interaction Effect: Developed SI Only by Timepoint	0.058	0.025	2.270	0.023	0.041	*
CBCL: Attention Problems	Interaction Effect: Endorsed SB by Timepoint	0.013	0.033	0.398	0.691	0.762	
CBCL: Attention Problems	Interaction Effect: Endorsed SI Only by Timepoint	0.009	0.017	0.518	0.604	0.689	
CBCL: Attention Problems	Interaction Effect: Developed SB by Timepoint	0.027	0.044	0.613	0.540	0.626	
**CBCL: Depression Syndrome**	**Interaction Effect: Developed SB by Timepoint**	**0.087**	**0.036**	**2.440**	**0.015**	**0.027**	*
CBCL: Depression Syndrome	Interaction Effect: Depressed Controls by Timepoint	0.036	0.039	0.931	0.352	0.441	
CBCL: Depression Syndrome	Interaction Effect: Developed SI Only by Timepoint	0.072	0.021	3.507	0.000	0.001	*
CBCL: Depression Syndrome	Interaction Effect: Endorsed SB by Timepoint	-0.016	0.027	-0.617	0.537	0.624	
CBCL: Depression Syndrome	Interaction Effect: Endorsed SI Only by Timepoint	-0.006	0.014	-0.471	0.638	0.717	
CBCL: DSM5 ADHD	Interaction Effect: Depressed Controls by Timepoint	0.010	0.052	0.192	0.847	0.886	
CBCL: DSM5 ADHD	Interaction Effect: Developed SI Only by Timepoint	0.060	0.028	2.169	0.030	0.052	

CBCL: DSM5 ADHD	Interaction Effect: Endorsed SB by Timepoint	0.010	0.036	0.288	0.773	0.832	
CBCL: DSM5 ADHD	Interaction Effect: Endorsed SI Only by Timepoint	0.004	0.018	0.224	0.823	0.868	
CBCL: DSM5 ADHD	Interaction Effect: Developed SB by Timepoint	0.024	0.048	0.503	0.615	0.697	
CBCL: DSM5 Anxiety Disorder	Interaction Effect: Depressed Controls by Timepoint	0.026	0.052	0.510	0.610	0.692	
CBCL: DSM5 Anxiety Disorder	Interaction Effect: Developed SI Only by Timepoint	0.065	0.027	2.369	0.018	0.032	*
CBCL: DSM5 Anxiety Disorder	Interaction Effect: Endorsed SB by Timepoint	-0.006	0.035	-0.167	0.867	0.901	
CBCL: DSM5 Anxiety Disorder	Interaction Effect: Endorsed SI Only by Timepoint	-0.011	0.018	-0.617	0.537	0.624	
CBCL: DSM5 Anxiety Disorder	Interaction Effect: Developed SB by Timepoint	0.049	0.047	1.029	0.304	0.390	
CBCL: DSM5 Conduct	Interaction Effect: Depressed Controls by Timepoint	0.038	0.031	1.200	0.230	0.311	
CBCL: DSM5 Conduct	Interaction Effect: Developed SI Only by Timepoint	0.032	0.017	1.945	0.052	0.086	
CBCL: DSM5 Conduct	Interaction Effect: Endorsed SB by Timepoint	-0.012	0.021	-0.579	0.563	0.649	
CBCL: DSM5 Conduct	Interaction Effect: Endorsed SI Only by Timepoint	-0.008	0.011	-0.757	0.449	0.533	
CBCL: DSM5 Conduct	Interaction Effect: Developed SB by Timepoint	0.031	0.028	1.093	0.274	0.359	
**CBCL: DSM5 Depression**	**Interaction Effect: Developed SB by Timepoint**	**0.154**	**0.036**	**4.268**	**0.000**	**0.000**	*
CBCL: DSM5 Depression	Interaction Effect: Depressed Controls by Timepoint	-0.032	0.040	-0.806	0.420	0.507	
CBCL: DSM5 Depression	Interaction Effect: Developed SI Only by Timepoint	0.052	0.021	2.493	0.013	0.024	*

CBCL: DSM5 Depression	Interaction Effect: Endorsed SB by Timepoint	-0.036	0.027	-1.310	0.190	0.267
CBCL: DSM5 Depression	Interaction Effect: Endorsed SI Only by Timepoint	0.000	0.014	0.024	0.981	0.986
CBCL: DSM5 Oppositional Defiance	Interaction Effect: Developed SB by Timepoint	0.075	0.051	1.488	0.137	0.202
CBCL: DSM5 Oppositional Defiance	Interaction Effect: Depressed Controls by Timepoint	-0.032	0.055	-0.571	0.568	0.653
CBCL: DSM5 Oppositional Defiance	Interaction Effect: Developed SI Only by Timepoint	0.032	0.029	1.110	0.267	0.353
CBCL: DSM5 Oppositional Defiance	Interaction Effect: Endorsed SB by Timepoint	-0.012	0.038	-0.323	0.747	0.809
CBCL: DSM5 Oppositional Defiance	Interaction Effect: Endorsed SI Only by Timepoint	-0.006	0.019	-0.316	0.752	0.814
CBCL: DSM5 Somatic Problems	Interaction Effect: Depressed Controls by Timepoint	0.017	0.050	0.343	0.732	0.798
CBCL: DSM5 Somatic Problems	Interaction Effect: Developed SI Only by Timepoint	-0.007	0.026	-0.275	0.784	0.839
CBCL: DSM5 Somatic Problems	Interaction Effect: Endorsed SB by Timepoint	-0.007	0.034	-0.204	0.839	0.880
CBCL: DSM5 Somatic Problems	Interaction Effect: Endorsed SI Only by Timepoint	0.003	0.017	0.161	0.872	0.904
CBCL: DSM5 Somatic Problems	Interaction Effect: Developed SB by Timepoint	0.007	0.045	0.165	0.869	0.902
CBCL: Externalizing	Interaction Effect: Developed SB by Timepoint	0.049	0.033	1.476	0.140	0.205
CBCL: Externalizing	Interaction Effect: Depressed Controls by Timepoint	0.005	0.036	0.140	0.889	0.917
CBCL: Externalizing	Interaction Effect: Developed SI Only by Timepoint	0.028	0.019	1.438	0.151	0.218
CBCL: Externalizing	Interaction Effect: Endorsed SB by Timepoint	-0.011	0.025	-0.428	0.669	0.744

CBCL: Externalizing	Interaction Effect: Endorsed SI Only by Timepoint	-0.012	0.013	-0.913	0.361	0.449	
CBCL: Internalizing	Interaction Effect: Developed SB by Timepoint	0.100	0.048	2.090	0.037	0.062	
CBCL: Internalizing	Interaction Effect: Depressed Controls by Timepoint	0.011	0.052	0.206	0.837	0.880	
CBCL: Internalizing	Interaction Effect: Developed SI Only by Timepoint	0.073	0.028	2.644	0.008	0.016	*
CBCL: Internalizing	Interaction Effect: Endorsed SB by Timepoint	-0.025	0.036	-0.687	0.492	0.576	
CBCL: Internalizing	Interaction Effect: Endorsed SI Only by Timepoint	-0.009	0.018	-0.477	0.633	0.714	
CBCL: OCD	Interaction Effect: Depressed Controls by Timepoint	-0.037	0.046	-0.799	0.424	0.510	
CBCL: OCD	Interaction Effect: Developed SI Only by Timepoint	0.031	0.024	1.282	0.200	0.277	
CBCL: OCD	Interaction Effect: Endorsed SB by Timepoint	-0.013	0.032	-0.412	0.681	0.753	
CBCL: OCD	Interaction Effect: Endorsed SI Only by Timepoint	-0.030	0.016	-1.846	0.065	0.104	
CBCL: OCD	Interaction Effect: Developed SB by Timepoint	0.072	0.042	1.721	0.085	0.133	
CBCL: Rule-Breaking	Interaction Effect: Developed SB by Timepoint	0.047	0.034	1.390	0.164	0.233	
CBCL: Rule-Breaking	Interaction Effect: Depressed Controls by Timepoint	0.054	0.037	1.444	0.149	0.216	
CBCL: Rule-Breaking	Interaction Effect: Developed SI Only by Timepoint	0.029	0.020	1.500	0.134	0.198	
CBCL: Rule-Breaking	Interaction Effect: Endorsed SB by Timepoint	-0.020	0.025	-0.770	0.442	0.526	
CBCL: Rule-Breaking	Interaction Effect: Endorsed SI Only by Timepoint	-0.008	0.013	-0.600	0.548	0.635	

CBCL: Sluggish Cognitive Tempo	Interaction Effect: Depressed Controls by Timepoint	0.005	0.036	0.138	0.890	0.917	
CBCL: Sluggish Cognitive Tempo	Interaction Effect: Developed SI Only by Timepoint	0.048	0.019	2.540	0.011	0.021	*
CBCL: Sluggish Cognitive Tempo	Interaction Effect: Endorsed SB by Timepoint	-0.006	0.024	-0.246	0.806	0.855	
CBCL: Sluggish Cognitive Tempo	Interaction Effect: Endorsed SI Only by Timepoint	0.001	0.012	0.076	0.940	0.950	
CBCL: Sluggish Cognitive Tempo	Interaction Effect: Developed SB by Timepoint	-0.042	0.033	-1.279	0.201	0.277	
CBCL: Social Problems	Interaction Effect: Depressed Controls by Timepoint	0.000	0.040	0.002	0.998	0.998	
CBCL: Social Problems	Interaction Effect: Developed SI Only by Timepoint	0.054	0.021	2.520	0.012	0.022	*
CBCL: Social Problems	Interaction Effect: Endorsed SB by Timepoint	-0.021	0.028	-0.779	0.436	0.522	
CBCL: Social Problems	Interaction Effect: Endorsed SI Only by Timepoint	0.007	0.014	0.510	0.610	0.692	
CBCL: Social Problems	Interaction Effect: Developed SB by Timepoint	0.004	0.037	0.113	0.910	0.932	
CBCL: Somatic Syndrome	Interaction Effect: Depressed Controls by Timepoint	0.004	0.050	0.079	0.937	0.949	
CBCL: Somatic Syndrome	Interaction Effect: Developed SI Only by Timepoint	-0.013	0.026	-0.481	0.630	0.711	
CBCL: Somatic Syndrome	Interaction Effect: Endorsed SB by Timepoint	-0.015	0.034	-0.450	0.653	0.729	
CBCL: Somatic Syndrome	Interaction Effect: Endorsed SI Only by Timepoint	0.013	0.017	0.776	0.438	0.523	
CBCL: Somatic Syndrome	Interaction Effect: Developed SB by Timepoint	0.029	0.045	0.639	0.523	0.610	
CBCL: Stress	Interaction Effect: Depressed Controls by Timepoint	0.004	0.050	0.078	0.938	0.949	

CBCL: Stress	Interaction Effect: Developed SI Only by Timepoint	0.060	0.026	2.259	0.024	0.042	*
CBCL: Stress	Interaction Effect: Endorsed SB by Timepoint	-0.024	0.034	-0.695	0.487	0.572	
CBCL: Stress	Interaction Effect: Endorsed SI Only by Timepoint	-0.007	0.017	-0.375	0.708	0.779	
CBCL: Stress	Interaction Effect: Developed SB by Timepoint	0.076	0.046	1.664	0.096	0.147	
CBCL: Thought Problems	Interaction Effect: Developed SB by Timepoint	0.073	0.039	1.871	0.061	0.099	
CBCL: Thought Problems	Interaction Effect: Depressed Controls by Timepoint	0.011	0.043	0.263	0.792	0.844	
CBCL: Thought Problems	Interaction Effect: Developed SI Only by Timepoint	0.000	0.023	0.019	0.985	0.988	
CBCL: Thought Problems	Interaction Effect: Endorsed SB by Timepoint	-0.024	0.029	-0.811	0.417	0.505	
CBCL: Thought Problems	Interaction Effect: Endorsed SI Only by Timepoint	0.016	0.015	1.068	0.285	0.370	
CBCL: Total Problems	Interaction Effect: Developed SB by Timepoint	0.067	0.057	1.169	0.242	0.325	
CBCL: Total Problems	Interaction Effect: Depressed Controls by Timepoint	0.050	0.062	0.806	0.420	0.507	
CBCL: Total Problems	Interaction Effect: Developed SI Only by Timepoint	0.096	0.033	2.903	0.004	0.007	*
CBCL: Total Problems	Interaction Effect: Endorsed SB by Timepoint	0.010	0.043	0.231	0.817	0.865	
CBCL: Total Problems	Interaction Effect: Endorsed SI Only by Timepoint	0.006	0.022	0.279	0.781	0.837	
**UPPS: Lack of Perserverance**	**Interaction Effect: Developed SB by Timepoint**	**0.315**	**0.107**	**2.933**	**0.003**	**0.007**	*
UPPS: Lack of Perserverance	Interaction Effect: Depressed Controls by Timepoint	0.113	0.123	0.916	0.360	0.448	

UPPS: Lack of Perserverance	Interaction Effect: Developed SI Only by Timepoint	0.308	0.063	4.882	0.000	0.000	*
UPPS: Lack of Perserverance	Interaction Effect: Endorsed SB by Timepoint	0.153	0.085	1.793	0.073	0.116	
UPPS: Lack of Perserverance	Interaction Effect: Endorsed SI Only by Timepoint	0.003	0.043	0.068	0.945	0.955	
**UPPS: Lack of Planning**	**Interaction Effect: Developed SB by Timepoint**	**0.405**	**0.108**	**3.757**	**0.000**	**0.000**	*
UPPS: Lack of Planning	Interaction Effect: Depressed Controls by Timepoint	0.119	0.124	0.957	0.338	0.428	
UPPS: Lack of Planning	Interaction Effect: Developed SI Only by Timepoint	0.246	0.063	3.884	0.000	0.000	*
UPPS: Lack of Planning	Interaction Effect: Endorsed SB by Timepoint	0.052	0.085	0.613	0.540	0.626	
UPPS: Lack of Planning	Interaction Effect: Endorsed SI Only by Timepoint	-0.051	0.043	-1.177	0.239	0.321	
**UPPS: Negative Urgency**	**Interaction Effect: Developed SB by Timepoint**	**0.268**	**0.112**	**2.381**	**0.017**	**0.032**	*
UPPS: Negative Urgency	Interaction Effect: Depressed Controls by Timepoint	0.108	0.129	0.835	0.404	0.491	
UPPS: Negative Urgency	Interaction Effect: Developed SI Only by Timepoint	0.366	0.066	5.524	0.000	0.000	*
UPPS: Negative Urgency	Interaction Effect: Endorsed SB by Timepoint	-0.172	0.089	-1.930	0.054	0.088	
UPPS: Negative Urgency	Interaction Effect: Endorsed SI Only by Timepoint	-0.093	0.045	-2.067	0.039	0.066	
UPPS: Positive Urgency	Interaction Effect: Developed SB by Timepoint	0.165	0.111	1.480	0.139	0.204	
UPPS: Positive Urgency	Interaction Effect: Depressed Controls by Timepoint	0.206	0.128	1.611	0.107	0.162	
UPPS: Positive Urgency	Interaction Effect: Developed SI Only by Timepoint	0.193	0.066	2.935	0.003	0.007	*

UPPS: Positive Urgency	Interaction Effect: Endorsed SB by Timepoint	-0.167	0.088	-1.888	0.059	0.096	
UPPS: Positive Urgency	Interaction Effect: Endorsed SI Only by Timepoint	-0.088	0.045	-1.982	0.048	0.079	
UPPS: Sensation Seeking	Interaction Effect: Developed SB by Timepoint	0.133	0.105	1.264	0.206	0.283	
UPPS: Sensation Seeking	Interaction Effect: Depressed Controls by Timepoint	0.012	0.122	0.096	0.923	0.941	
UPPS: Sensation Seeking	Interaction Effect: Developed SI Only by Timepoint	0.141	0.062	2.293	0.022	0.039	*
UPPS: Sensation Seeking	Interaction Effect: Endorsed SB by Timepoint	-0.104	0.083	-1.247	0.212	0.291	
UPPS: Sensation Seeking	Interaction Effect: Endorsed SI Only by Timepoint	-0.022	0.042	-0.517	0.605	0.689	

Supplementary Table 10 - Comparison of *Group by Timepoint* interaction effects representing differences in the rates of developmental change in behavioral and psychiatric measures between specified groups. These comparisons were assessed using general linear hypotheses post-hoc contrasts which tests the null hypothesis of no significant difference between two specified *group by timepoint* interaction effects (i.e. Developed SB by Timepoint - Developed SI by Timepoint = 0).

Psychiatric/Behavioral Measure	Contrasts	Estimate	Standard Error	Z-value	P-value (adjusted)	Significance
CBCL: Anxious Depression Syndrome	Developed SB by Timepoint - Developed SI Only by Timepoint = 0	0.04	0.05	0.91	0.40	
CBCL: Depression Syndrome	Developed SB by Timepoint - Developed SI Only by Timepoint = 0	0.01	0.04	0.40	0.76	
CBCL: DSM5 Depression	Developed SB by Timepoint - Developed SI Only by Timepoint = 0	0.10	0.04	2.72	0.01	*
UPPS: Negative Urgency	Developed SB by Timepoint - Developed SI Only by Timepoint = 0	-0.10	0.12	-0.81	0.46	
UPPS: Lack of Planning	Developed SB by Timepoint - Developed SI Only by Timepoint = 0	0.16	0.11	1.39	0.22	
UPPS: Lack of Perserverance	Developed SB by Timepoint - Developed SI Only by Timepoint = 0	0.01	0.11	0.06	0.95	
BIS/BAS: Fun Seeking	Developed SB by Timepoint - Endorsed SI Only by Timepoint = 0	0.36	0.11	3.20	0.00	*

BIS/BAS: Fun Seeking	Developed SB by Timepoint - Endorsed SB by Timepoint = 0	0.52	0.13	3.92	0.00	*

Supplementary Table 11 - Assessment of estimated differences in behavioral and psychiatric measures between those who developed SB and other groups at each timepoint using general linear hypotheses post-hoc contrasts which tests the null hypothesis of no significant difference in these measures between two specified groups at a specified timepoint (ex. Developed SB - Developed SI at Baseline = 0). Note, contrasts between those who developed SB and other non-depressed controls at baseline were already estimated as main effects in the original regression models and were not included here.

Psychiatric/Behavioral Measure	Contrasts	Estimate	Standard Error	Z-value	P-value (adjusted)	Significance
CBCL: Anxious Depression Syndrome	Developed SB - Developed SI Only at Baseline = 0	0.116	0.040	2.881	0.007	*
CBCL: Anxious Depression Syndrome	Developed SB - Endorsed SB at Baseline = 0	-0.022	0.044	-0.502	0.615	
CBCL: Anxious Depression Syndrome	Developed SB - Endorsed SI Only at Baseline = 0	0.100	0.037	2.678	0.012	*
CBCL: Anxious Depression Syndrome	Developed SB - Depressed Controls at Baseline = 0	-0.379	0.052	-7.308	0.000	*
CBCL: Anxious Depression Syndrome	Developed SB - Developed SI Only at Two-Year Follow-Up = 0	0.157	0.046	3.401	0.001	*
CBCL: Anxious Depression Syndrome	Developed SB - Endorsed SB at Two-Year Follow-Up = 0	0.118	0.051	2.334	0.027	*
CBCL: Anxious Depression Syndrome	Developed SB - Endorsed SI Only at Two-Year Follow-Up = 0	0.217	0.043	5.058	0.000	*
CBCL: Anxious Depression Syndrome	Developed SB - Depressed Controls at Two-Year Follow-Up = 0	-0.270	0.061	-4.455	0.000	*
CBCL: Anxious Depression Syndrome	Developed SB - Non-Depressed Controls at Two-Year Follow-Up = 0	0.350	0.041	8.588	0.000	*
CBCL: Depression Syndrome	Developed SB - Developed SI Only at Baseline = 0	0.152	0.032	4.805	0.000	*
CBCL: Depression Syndrome	Developed SB - Endorsed SB at Baseline = 0	0.002	0.035	0.045	0.964	
CBCL: Depression Syndrome	Developed SB - Endorsed SI Only at Baseline = 0	0.115	0.029	3.945	0.000	*

CBCL: Depression Syndrome	Developed SB - Depressed Controls at Baseline = 0	-0.385	0.041	-9.479	0.000	*
CBCL: Depression Syndrome	Developed SB - Developed SI Only at Two-Year Follow-Up = 0	0.166	0.037	4.554	0.000	*
CBCL: Depression Syndrome	Developed SB - Endorsed SB at Two-Year Follow-Up = 0	0.105	0.040	2.628	0.011	*
CBCL: Depression Syndrome	Developed SB - Endorsed SI Only at Two-Year Follow-Up = 0	0.208	0.034	6.164	0.000	*
CBCL: Depression Syndrome	Developed SB - Depressed Controls at Two-Year Follow-Up = 0	-0.335	0.048	-6.986	0.000	*
CBCL: Depression Syndrome	Developed SB - Non-Depressed Controls at Two-Year Follow-Up = 0	0.300	0.032	9.331	0.000	*
CBCL: DSM5 Depression	Developed SB - Developed SI Only at Baseline = 0	0.088	0.032	2.801	0.006	*
CBCL: DSM5 Depression	Developed SB - Endorsed SB at Baseline = 0	-0.059	0.035	-1.713	0.087	
CBCL: DSM5 Depression	Developed SB - Endorsed SI Only at Baseline = 0	0.092	0.029	3.140	0.002	*
CBCL: DSM5 Depression	Developed SB - Depressed Controls at Baseline = 0	-0.548	0.041	-13.488	0.000	*
CBCL: DSM5 Depression	Developed SB - Developed SI Only at Two-Year Follow-Up = 0	0.190	0.037	5.194	0.000	*
CBCL: DSM5 Depression	Developed SB - Endorsed SB at Two-Year Follow-Up = 0	0.130	0.040	3.255	0.002	*
CBCL: DSM5 Depression	Developed SB - Endorsed SI Only at Two-Year Follow-Up = 0	0.245	0.034	7.236	0.000	*
CBCL: DSM5 Depression	Developed SB - Depressed Controls at Two-Year Follow-Up = 0	-0.362	0.048	-7.530	0.000	*
CBCL: DSM5 Depression	Developed SB - Non-Depressed Controls at Two-Year Follow-Up = 0	0.362	0.032	11.242	0.000	*
UPPS: Negative Urgency	Developed SB - Developed SI Only at Baseline = 0	0.379	0.097	3.910	0.000	*
UPPS: Negative Urgency	Developed SB - Endorsed SB at Baseline = 0	-0.242	0.107	-2.264	0.035	*

UPPS: Negative Urgency	Developed SB - Endorsed SI Only at Baseline = 0	0.145	0.090	1.616	0.127	
UPPS: Negative Urgency	Developed SB - Depressed Controls at Baseline = 0	0.469	0.125	3.757	0.000	*
UPPS: Negative Urgency	Developed SB - Developed SI Only at Two-Year Follow-Up = 0	0.281	0.101	2.779	0.009	*
UPPS: Negative Urgency	Developed SB - Endorsed SB at Two-Year Follow-Up = 0	0.198	0.112	1.776	0.101	
UPPS: Negative Urgency	Developed SB - Endorsed SI Only at Two-Year Follow-Up = 0	0.506	0.094	5.406	0.000	*
UPPS: Negative Urgency	Developed SB - Depressed Controls at Two-Year Follow-Up = 0	0.629	0.136	4.605	0.000	*
UPPS: Negative Urgency	Developed SB - Non-Depressed Controls at Two-Year Follow-Up = 0	0.866	0.088	9.822	0.000	*
UPPS: Lack of Planning	Developed SB - Developed SI Only at Baseline = 0	0.047	0.099	0.472	0.637	
UPPS: Lack of Planning	Developed SB - Endorsed SB at Baseline = 0	-0.263	0.109	-2.410	0.044	*
UPPS: Lack of Planning	Developed SB - Endorsed SI Only at Baseline = 0	-0.167	0.092	-1.822	0.125	
UPPS: Lack of Planning	Developed SB - Depressed Controls at Baseline = 0	-0.077	0.128	-0.607	0.598	
UPPS: Lack of Planning	Developed SB - Developed SI Only at Two-Year Follow-Up = 0	0.206	0.103	1.999	0.100	
UPPS: Lack of Planning	Developed SB - Endorsed SB at Two-Year Follow-Up = 0	0.090	0.114	0.793	0.523	
UPPS: Lack of Planning	Developed SB - Endorsed SI Only at Two-Year Follow-Up = 0	0.289	0.095	3.031	0.009	*
UPPS: Lack of Planning	Developed SB - Depressed Controls at Two-Year Follow-Up = 0	0.209	0.139	1.510	0.206	
UPPS: Lack of Planning	Developed SB - Non-Depressed Controls at Two-Year Follow-Up = 0	0.614	0.090	6.838	0.000	*
UPPS: Lack of Perserverance	Developed SB - Developed SI Only at Baseline = 0	0.230	0.099	2.328	0.046	*

UPPS: Lack of Perserverance	Developed SB - Endorsed SB at Baseline = 0	-0.047	0.109	-0.428	0.784	
UPPS: Lack of Perserverance	Developed SB - Endorsed SI Only at Baseline = 0	0.090	0.092	0.980	0.450	
UPPS: Lack of Perserverance	Developed SB - Depressed Controls at Baseline = 0	0.047	0.127	0.368	0.784	
UPPS: Lack of Perserverance	Developed SB - Developed SI Only at Two-Year Follow-Up = 0	0.237	0.103	2.307	0.046	*
UPPS: Lack of Perserverance	Developed SB - Endorsed SB at Two-Year Follow-Up = 0	0.116	0.113	1.021	0.450	
UPPS: Lack of Perserverance	Developed SB - Endorsed SI Only at Two-Year Follow-Up = 0	0.402	0.095	4.229	0.000	*
UPPS: Lack of Perserverance	Developed SB - Depressed Controls at Two-Year Follow-Up = 0	0.249	0.138	1.801	0.132	
UPPS: Lack of Perserverance	Developed SB - Non-Depressed Controls at Two-Year Follow-Up = 0	0.708	0.090	7.900	0.000	*
BIS/BAS: BIS Sum	Developed SB - Developed SI Only at Baseline = 0	0.302	0.098	3.079	0.003	*
BIS/BAS: BIS Sum	Developed SB - Endorsed SB at Baseline = 0	0.122	0.108	1.134	0.285	
BIS/BAS: BIS Sum	Developed SB - Endorsed SI Only at Baseline = 0	0.267	0.091	2.944	0.005	*
BIS/BAS: BIS Sum	Developed SB - Depressed Controls at Baseline = 0	0.490	0.126	3.884	0.000	*
BIS/BAS: BIS Sum	Developed SB - Developed SI Only at Two-Year Follow-Up = 0	0.205	0.102	2.012	0.055	
BIS/BAS: BIS Sum	Developed SB - Endorsed SB at Two-Year Follow-Up = 0	0.466	0.113	4.134	0.000	*
BIS/BAS: BIS Sum	Developed SB - Endorsed SI Only at Two-Year Follow-Up = 0	0.480	0.094	5.082	0.000	*
BIS/BAS: BIS Sum	Developed SB - Depressed Controls at Two-Year Follow-Up = 0	0.470	0.137	3.420	0.001	*
BIS/BAS: BIS Sum	Developed SB - Non-Depressed Controls at Two-Year Follow-Up = 0	0.819	0.089	9.207	0.000	*

BIS/BAS: Fun Seeking	Developed SB - Developed SI Only at Baseline = 0	0.067	0.098	0.685	0.538	
BIS/BAS: Fun Seeking	Developed SB - Endorsed SB at Baseline = 0	-0.326	0.108	-3.025	0.005	*
BIS/BAS: Fun Seeking	Developed SB - Endorsed SI Only at Baseline = 0	-0.029	0.091	-0.315	0.753	
BIS/BAS: Fun Seeking	Developed SB - Depressed Controls at Baseline = 0	0.262	0.126	2.077	0.057	
BIS/BAS: Fun Seeking	Developed SB - Developed SI Only at Two-Year Follow-Up = 0	0.225	0.102	2.204	0.047	*
BIS/BAS: Fun Seeking	Developed SB - Endorsed SB at Two-Year Follow-Up = 0	0.198	0.113	1.758	0.105	
BIS/BAS: Fun Seeking	Developed SB - Endorsed SI Only at Two-Year Follow-Up = 0	0.330	0.094	3.494	0.002	*
BIS/BAS: Fun Seeking	Developed SB - Depressed Controls at Two-Year Follow-Up = 0	0.419	0.138	3.040	0.005	*
BIS/BAS: Fun Seeking	Developed SB - Non-Depressed Controls at Two-Year Follow-Up = 0	0.418	0.089	4.693	0.000	*

Supplementary Table 12 - Total estimated change in behavioral and psychiatric measures between 10 to 12 years of age for a specified group assessed using general linear hypotheses post-hoc contrasts which tests the null hypothesis of no significant total effect of timepoint and group by timepoint interaction effects combined (i.e. Timepoint + Developed SB by Timepoint = 0).

Psychiatric/Behavioral Measure	Contrasts	Estimate	Standard Error	Z-value	P-value (adjusted)	Significance
CBCL: Anxious Depression Syndrome	Timepoint + Developed SB by Timepoint = 0	0.05	0.04	1.20	0.28	
CBCL: Depression Syndrome	Timepoint + Developed SB by Timepoint = 0	0.10	0.03	3.21	0.00	*
CBCL: DSM5 Depression	Timepoint + Developed SB by Timepoint = 0	0.17	0.03	5.28	0.00	*
UPPS: Negative Urgency	Timepoint + Developed SB by Timepoint = 0	-0.04	0.11	-0.39	0.69	
UPPS: Lack of Planning	Timepoint + Developed SB by Timepoint = 0	0.39	0.10	3.89	0.00	*
UPPS: Lack of Perserverance	Timepoint + Developed SB by Timepoint = 0	0.23	0.10	2.31	0.05	*
BIS/BAS: BIS Sum	Timepoint + Developed SI Only by Timepoint = 0	0.04	0.06	0.62	0.53	
BIS/BAS: Fun Seeking	Timepoint + Developed SB by Timepoint = 0	-0.15	0.11	-1.44	0.18	

Supplementary Table 13 - Testing the Significance of Cross-Lagged Causal Paths Relating Total Cortical Gray Matter Volume and BAS Fun-Seeking Scores.

Base Model	Comaprison Model	Estimated Parameters	minus2Log-Likelihood	Degrees of Freedom	AIC	Difference in Log-Likelihood	Difference in Degrees of Freedom	p-value
Full CLPM Model	NA	14	70282.986	29190	70310.986	NA	NA	NA
Full CLPM Model	Alternative Model: No BAS Fun-Seeking to Cortical Gray Matter Volume Cross-Lagged Path	13	70283.133	29191	70309.133	0.147	1	0.701
Full CLPM Model	Alternative Model: No Cortical Gray Matter Volume to BAS Fun-Seeking Cross-Lagged Path	13	70287.847	29191	70313.847	4.861	1	0.027

Supplementary Table 14 - Testing the Significance of Cross-Lagged Causal Paths Relating Total Cortical Gray Matter Volume and DSM5 Depression Scores.

Base Model	Comaprison Model	Estimated Parameters	minus2Log-Likelihood	Degrees of Freedom	AIC	Difference in Log-Likelihood	Difference in Degrees of Freedom	p-value
Full CLPM Model	NA	14	69520.621	29190	69548.621	NA	NA	NA
Full CLPM Model	Alternative Model: No DSM5 Depression Score to Cortical Gray Matter Volume Cross-Lagged Path	13	69520.629	29191	69546.629	0.008	1	0.929
Full CLPM Model	Alternative Model: No Cortical Gray Matter Volume to DSM5 Depression Cross-Lagged Path	13	69521.376	29191	69547.376	0.755	1	0.385

Supplementary Table 15 - Main effects of SES indicators including poverty, combined family income, and parental education measures from multiple linear mixed effects regression for 9 global brain volume measures. The effect estimates represent how different levels of SES indicators impact brain volume. The reference groups for the poverty, combined family income, and parental education indicators were the no poverty indicators, less than 50K per year, and high school or less groups, respectively.

sMRI Measures (outcome vari	Regression Parameters (predictor varia	Estimate	Std. Error	t value	p-value	p.adjusted
Total Brain Volume	Main Effect: Poverty - One Indicator	-6.36	0.67	-9.50	0.00	0.00
Total Brain Volume	Main Effect: Poverty - Two Indicators or More	-8.14	0.61	-13.42	0.00	0.00
Total Brain Volume	Main Effect: Combined Family Income - Between 50K to 100K	7.51	0.55	13.59	0.00	0.00
Total Brain Volume	Main Effect: Combined Family Income - Above 100K	16.33	0.60	27.04	0.00	0.00
Total Brain Volume	Main Effect: Parental Education - Post-Secondary but No College	6.73	0.70	9.57	0.00	0.00
Total Brain Volume	Main Effect: Parental Education - Undergraduate or Above	18.85	0.76	24.77	0.00	0.00
Total Cortical Gray Matter Volume	Main Effect: Poverty - One Indicator	-3.22	0.48	-6.66	0.00	0.00
Total Cortical Gray Matter Volume	Main Effect: Poverty - Two Indicators or More	-4.69	0.44	-10.73	0.00	0.00
Total Cortical Gray Matter Volume	Main Effect: Combined Family Income - Between 50K to 100K	3.63	0.39	9.36	0.00	0.00
Total Cortical Gray Matter Volume	Main Effect: Combined Family Income - Above 100K	8.27	0.43	19.20	0.00	0.00
Total Cortical Gray Matter Volume	Main Effect: Parental Education - Post-Secondary but No College	3.34	0.50	6.70	0.00	0.00
Total Cortical Gray Matter Volume	Main Effect: Parental Education - Undergraduate or Above	10.46	0.54	19.37	0.00	0.00
Total Left Cortical White Matter Volume	Main Effect: Poverty - One Indicator	-1.33	0.15	-8.82	0.00	0.00

Total Left Cortical White Matter Volume	Main Effect: Poverty - Two Indicators or More	-1.35	0.14	-9.97	0.00	0.00
Total Left Cortical White Matter Volume	Main Effect: Combined Family Income - Between 50K to 100K	1.11	0.12	9.14	0.00	0.00
Total Left Cortical White Matter Volume	Main Effect: Combined Family Income - Above 100K	2.67	0.13	19.83	0.00	0.00
Total Left Cortical White Matter Volume	Main Effect: Parental Education - Post-Secondary but No College	1.20	0.16	7.68	0.00	0.00
Total Left Cortical White Matter Volume	Main Effect: Parental Education - Undergraduate or Above	2.62	0.17	15.64	0.00	0.00
Total Right Cortical White Matter Volume	Main Effect: Poverty - One Indicator	-1.24	0.15	-8.03	0.00	0.00
Total Right Cortical White Matter Volume	Main Effect: Poverty - Two Indicators or More	-1.38	0.14	-10.09	0.00	0.00
Total Right Cortical White Matter Volume	Main Effect: Combined Family Income - Between 50K to 100K	1.09	0.13	8.70	0.00	0.00
Total Right Cortical White Matter Volume	Main Effect: Combined Family Income - Above 100K	2.63	0.14	19.08	0.00	0.00
Total Right Cortical White Matter Volume	Main Effect: Parental Education - Post-Secondary but No College	1.05	0.16	6.52	0.00	0.00
Total Right Cortical White Matter Volume	Main Effect: Parental Education - Undergraduate or Above	2.51	0.17	14.51	0.00	0.00
Total Left Cerebellar Cortex Volume	Main Effect: Poverty - One Indicator	-0.23	0.05	-4.46	0.00	0.00
Total Left Cerebellar Cortex Volume	Main Effect: Poverty - Two Indicators or More	-0.49	0.05	-10.71	0.00	0.00
Total Left Cerebellar Cortex Volume	Main Effect: Combined Family Income - Between 50K to 100K	0.37	0.04	8.90	0.00	0.00
Total Left Cerebellar Cortex Volume	Main Effect: Combined Family Income - Above 100K	0.69	0.04	15.43	0.00	0.00
Total Left Cerebellar Cortex Volume	Main Effect: Parental Education - Post-Secondary but No College	0.48	0.05	9.04	0.00	0.00

Total Left Cerebellar Cortex Volume	Main Effect: Parental Education - Undergraduate or Above	1.21	0.06	20.77	0.00	0.00
Total Right Cerebellar Cortex Volume	Main Effect: Poverty - One Indicator	-0.20	0.06	-3.48	0.00	0.00
Total Right Cerebellar Cortex Volume	Main Effect: Poverty - Two Indicators or More	-0.43	0.05	-8.24	0.00	0.00
Total Right Cerebellar Cortex Volume	Main Effect: Combined Family Income - Between 50K to 100K	0.37	0.05	7.67	0.00	0.00
Total Right Cerebellar Cortex Volume	Main Effect: Combined Family Income - Above 100K	0.73	0.05	14.10	0.00	0.00
Total Right Cerebellar Cortex Volume	Main Effect: Parental Education - Post-Secondary but No College	0.53	0.06	8.71	0.00	0.00
Total Right Cerebellar Cortex Volume	Main Effect: Parental Education - Undergraduate or Above	1.23	0.07	18.71	0.00	0.00
Total Left Cerebellar White Matter Volume	Main Effect: Poverty - One Indicator	-0.08	0.04	-2.22	0.03	0.04
Total Left Cerebellar White Matter Volume	Main Effect: Poverty - Two Indicators or More	-0.11	0.03	-3.23	0.00	0.00
Total Left Cerebellar White Matter Volume	Main Effect: Combined Family Income - Between 50K to 100K	0.13	0.03	4.32	0.00	0.00
Total Left Cerebellar White Matter Volume	Main Effect: Combined Family Income - Above 100K	0.22	0.03	6.76	0.00	0.00
Total Left Cerebellar White Matter Volume	Main Effect: Parental Education - Post-Secondary but No College	0.03	0.04	0.71	0.48	0.52
Total Left Cerebellar White Matter Volume	Main Effect: Parental Education - Undergraduate or Above	0.15	0.04	3.73	0.00	0.00
Total Right Cerebellar White Matter Volume	Main Effect: Poverty - One Indicator	-0.02	0.03	-0.62	0.53	0.58
Total Right Cerebellar White Matter Volume	Main Effect: Poverty - Two Indicators or More	-0.12	0.03	-4.21	0.00	0.00
Total Right Cerebellar White Matter Volume	Main Effect: Combined Family Income - Between 50K to 100K	0.10	0.03	3.86	0.00	0.00

Total Right Cerebellar White Matter Volume	Main Effect: Combined Family Income - Above 100K	0.19	0.03	6.54	0.00	0.00
Total Right Cerebellar White Matter Volume	Main Effect: Parental Education - Post-Secondary but No College	0.07	0.03	1.91	0.06	0.08
Total Right Cerebellar White Matter Volume	Main Effect: Parental Education - Undergraduate or Above	0.17	0.04	4.66	0.00	0.00
Total Subcortical Gray Matter Volume	Main Effect: Poverty - One Indicator	-0.33	0.04	-9.16	0.00	0.00
Total Subcortical Gray Matter Volume	Main Effect: Poverty - Two Indicators or More	-0.19	0.03	-5.97	0.00	0.00
Total Subcortical Gray Matter Volume	Main Effect: Combined Family Income - Between 50K to 100K	0.43	0.03	14.61	0.00	0.00
Total Subcortical Gray Matter Volume	Main Effect: Combined Family Income - Above 100K	0.74	0.03	22.60	0.00	0.00
Total Subcortical Gray Matter Volume	Main Effect: Parental Education - Post-Secondary but No College	0.31	0.04	8.30	0.00	0.00
Total Subcortical Gray Matter Volume	Main Effect: Parental Education - Undergraduate or Above	0.73	0.04	17.88	0.00	0.00

Supplementary Table 16 - Number of outliers removed for each global brain volume measure.

sMRI measure	Outliers Removed (n)	Percentage Removed (%)
Total Brain Volume	117	0.49
Total Cortical Gray Matter Volume	124	0.52
Total Left Cortical White Matter Volume	162	0.68
Total Right Cortical White Matter Volume	161	0.68
Total Left Cerebellar Cortex Volume	178	0.75
Total Right Cerebellar Cortex Volume	172	0.72
Total Left Cerebellar White Matter Volume	226	0.95
Total Right Cerebellar White Matter Volume	282	1.19
Total Subcortical Gray Matter Volume	149	0.63

Supplementary Table 17 - Intraclass Correlations for Nested Random Effects for Regressions involving Psychiatric and Behavioral Data.

sMRI	random_effect	ICC
CBCL: Anxious Depression Syndrome	site:rel_family_id:subjectkey	0.24
CBCL: Anxious Depression Syndrome	site:rel_family_id	0.29
CBCL: Anxious Depression Syndrome	site	0.01
CBCL: Anxious Depression Syndrome	Residual	0.46
CBCL: Depression Syndrome	site:rel_family_id:subjectkey	0.29
CBCL: Depression Syndrome	site:rel_family_id	0.20
CBCL: Depression Syndrome	site	0.01
CBCL: Depression Syndrome	Residual	0.50
CBCL: Somatic Syndrome	site:rel_family_id:subjectkey	0.14
CBCL: Somatic Syndrome	site:rel_family_id	0.32
CBCL: Somatic Syndrome	site	0.01
CBCL: Somatic Syndrome	Residual	0.53
CBCL: Social Problems	site:rel_family_id:subjectkey	0.27
CBCL: Social Problems	site:rel_family_id	0.27
CBCL: Social Problems	site	0.01
CBCL: Social Problems	Residual	0.46
CBCL: Thought Problems	site:rel_family_id:subjectkey	0.22
CBCL: Thought Problems	site:rel_family_id	0.30
CBCL: Thought Problems	site	0.01
CBCL: Thought Problems	Residual	0.47
CBCL: Attention Problems	site:rel_family_id:subjectkey	0.40
CBCL: Attention Problems	site:rel_family_id	0.23
CBCL: Attention Problems	site	0.01
CBCL: Attention Problems	Residual	0.35
CBCL: Rule-Breaking	site:rel_family_id:subjectkey	0.20
CBCL: Rule-Breaking	site:rel_family_id	0.33
CBCL: Rule-Breaking	site	0.01
CBCL: Rule-Breaking	Residual	0.47
CBCL: Aggressive Syndrome	site:rel_family_id:subjectkey	0.29
CBCL: Aggressive Syndrome	site:rel_family_id	0.35
CBCL: Aggressive Syndrome	site	0.01
CBCL: Aggressive Syndrome	Residual	0.35
CBCL: Internalizing	site:rel_family_id:subjectkey	0.19
CBCL: Internalizing	site:rel_family_id	0.36
CBCL: Internalizing	site	0.01
CBCL: Internalizing	Residual	0.43
CBCL: Externalizing	site:rel_family_id:subjectkey	0.27
CBCL: Externalizing	site:rel_family_id	0.37
CBCL: Externalizing	site	0.01

CBCL: Externalizing	Residual	0.35
CBCL: Total Problems	site:rel_family_id:subjectkey	0.15
CBCL: Total Problems	site:rel_family_id	0.44
CBCL: Total Problems	site	0.02
CBCL: Total Problems	Residual	0.40
CBCL: DSM5 Depression	site:rel_family_id:subjectkey	0.20
CBCL: DSM5 Depression	site:rel_family_id	0.27
CBCL: DSM5 Depression	site	0.01
CBCL: DSM5 Depression	Residual	0.52
CBCL: DSM5 Anxiety Disorder	site:rel_family_id:subjectkey	0.24
CBCL: DSM5 Anxiety Disorder	site:rel_family_id	0.29
CBCL: DSM5 Anxiety Disorder	site	0.01
CBCL: DSM5 Anxiety Disorder	Residual	0.46
CBCL: DSM5 Somatic Problems	site:rel_family_id:subjectkey	0.15
CBCL: DSM5 Somatic Problems	site:rel_family_id	0.29
CBCL: DSM5 Somatic Problems	site	0.00
CBCL: DSM5 Somatic Problems	Residual	0.56
CBCL: DSM5 ADHD	site:rel_family_id:subjectkey	0.37
CBCL: DSM5 ADHD	site:rel_family_id	0.25
CBCL: DSM5 ADHD	site	0.01
CBCL: DSM5 ADHD	Residual	0.37
CBCL: DSM5 Oppositional Defiance	site:rel_family_id:subjectkey	0.29
CBCL: DSM5 Oppositional Defiance	site:rel_family_id	0.30
CBCL: DSM5 Oppositional Defiance	site	0.01
CBCL: DSM5 Oppositional Defiance	Residual	0.41
CBCL: DSM5 Conduct	site:rel_family_id:subjectkey	0.25
CBCL: DSM5 Conduct	site:rel_family_id	0.32
CBCL: DSM5 Conduct	site	0.01
CBCL: DSM5 Conduct	Residual	0.43
CBCL: Sluggish Cognitive Tempo	site:rel_family_id:subjectkey	0.37
CBCL: Sluggish Cognitive Tempo	site:rel_family_id	0.13
CBCL: Sluggish Cognitive Tempo	site	0.01
CBCL: Sluggish Cognitive Tempo	Residual	0.49
CBCL: OCD	site:rel_family_id:subjectkey	0.25
CBCL: OCD	site:rel_family_id	0.25
CBCL: OCD	site	0.01
CBCL: OCD	Residual	0.49
CBCL: Stress	site:rel_family_id:subjectkey	0.22
CBCL: Stress	site:rel_family_id	0.33
CBCL: Stress	site	0.01
CBCL: Stress	Residual	0.43
UPPS: Negative Urgency	site:rel_family_id:subjectkey	0.14
UPPS: Negative Urgency	site:rel_family_id	0.12
UPPS: Negative Urgency	site	0.01

UPPS: Negative Urgency	Residual	0.74
UPPS: Lack of Planning	site:rel_family_id:subjectkey	0.27
UPPS: Lack of Planning	site:rel_family_id	0.11
UPPS: Lack of Planning	site	0.00
UPPS: Lack of Planning	Residual	0.62
UPPS: Sensation Seeking	site:rel_family_id:subjectkey	0.26
UPPS: Sensation Seeking	site:rel_family_id	0.17
UPPS: Sensation Seeking	site	0.01
UPPS: Sensation Seeking	Residual	0.57
UPPS: Positive Urgency	site:rel_family_id:subjectkey	0.16
UPPS: Positive Urgency	site:rel_family_id	0.12
UPPS: Positive Urgency	site	0.00
UPPS: Positive Urgency	Residual	0.72
UPPS: Lack of Perserverance	site:rel_family_id:subjectkey	0.21
UPPS: Lack of Perserverance	site:rel_family_id	0.17
UPPS: Lack of Perserverance	site	0.00
UPPS: Lack of Perserverance	Residual	0.62
BIS/BAS: BIS Sum Score	site:rel_family_id:subjectkey	0.20
BIS/BAS: BIS Sum Score	site:rel_family_id	0.12
BIS/BAS: BIS Sum Score	site	0.01
BIS/BAS: BIS Sum Score	Residual	0.67
BIS/BAS: Reward Responsiveness	site:rel_family_id:subjectkey	0.14
BIS/BAS: Reward Responsiveness	site:rel_family_id	0.14
BIS/BAS: Reward Responsiveness	site	0.01
BIS/BAS: Reward Responsiveness	Residual	0.71
BIS/BAS: Drive	site:rel_family_id:subjectkey	0.13
BIS/BAS: Drive	site:rel_family_id	0.16
BIS/BAS: Drive	site	0.01
BIS/BAS: Drive	Residual	0.70
BIS/BAS: Fun Seeking	site:rel_family_id:subjectkey	0.18
BIS/BAS: Fun Seeking	site:rel_family_id	0.09
BIS/BAS: Fun Seeking	site	0.01
BIS/BAS: Fun Seeking	Residual	0.72
BIS/BAS: BIS Sum Score Modified	site:rel_family_id:subjectkey	0.19
BIS/BAS: BIS Sum Score Modified	site:rel_family_id	0.11
BIS/BAS: BIS Sum Score Modified	site	0.00
BIS/BAS: BIS Sum Score Modified	Residual	0.69
BIS/BAS: Reward Responsiveness Modified	site:rel_family_id:subjectkey	0.14
BIS/BAS: Reward Responsiveness Modified	site:rel_family_id	0.13
BIS/BAS: Reward Responsiveness Modified	site	0.01
BIS/BAS: Reward Responsiveness Modified	Residual	0.73
BIS/BAS: Drive Modified	site:rel_family_id:subjectkey	0.13
BIS/BAS: Drive Modified	site:rel_family_id	0.16
BIS/BAS: Drive Modified	site	0.01

BIS/BAS: Drive Modified	Residual	0.70

Supplementary Table 18 - Intraclass Correlations for Nested Random Effects for Regressions Involving Global Brain Volume Measures.

sMRI	random_effect	ICC
Total Brain Volume	site:rel_family_id:subjectkey	0.39
Total Brain Volume	site:rel_family_id	0.58
Total Brain Volume	site	0.02
Total Brain Volume	Residual	0.01
Total Cortical Gray Matter Volume	site:rel_family_id:subjectkey	0.38
Total Cortical Gray Matter Volume	site:rel_family_id	0.56
Total Cortical Gray Matter Volume	site	0.02
Total Cortical Gray Matter Volume	Residual	0.03
Total Left Cortical White Matter Volume	site:rel_family_id:subjectkey	0.41
Total Left Cortical White Matter Volume	site:rel_family_id	0.57
Total Left Cortical White Matter Volume	site	0.01
Total Left Cortical White Matter Volume	Residual	0.01
Total Right Cortical White Matter Volume	site:rel_family_id:subjectkey	0.41
Total Right Cortical White Matter Volume	site:rel_family_id	0.57
Total Right Cortical White Matter Volume	site	0.01
Total Right Cortical White Matter Volume	Residual	0.01
Total Left Cerebellar Cortex Volume	site:rel_family_id:subjectkey	0.40
Total Left Cerebellar Cortex Volume	site:rel_family_id	0.55
Total Left Cerebellar Cortex Volume	site	0.02
Total Left Cerebellar Cortex Volume	Residual	0.03
Total Right Cerebellar Cortex Volume	site:rel_family_id:subjectkey	0.41
Total Right Cerebellar Cortex Volume	site:rel_family_id	0.52
Total Right Cerebellar Cortex Volume	site	0.03
Total Right Cerebellar Cortex Volume	Residual	0.04
Total Left Cerebellar White Matter Volume	site:rel_family_id:subjectkey	0.32
Total Left Cerebellar White Matter Volume	site:rel_family_id	0.45
Total Left Cerebellar White Matter Volume	site	0.07
Total Left Cerebellar White Matter Volume	Residual	0.16
Total Right Cerebellar White Matter Volume	site:rel_family_id:subjectkey	0.32
Total Right Cerebellar White Matter Volume	site:rel_family_id	0.47
Total Right Cerebellar White Matter Volume	site	0.06
Total Right Cerebellar White Matter Volume	Residual	0.15
Total Subcortical Gray Matter Volume	site:rel_family_id:subjectkey	0.41
Total Subcortical Gray Matter Volume	site:rel_family_id	0.56
Total Subcortical Gray Matter Volume	site	0.01
Total Subcortical Gray Matter Volume	Residual	0.02

Supplementary Table 19 - distribuation of study participants classified as those who have *endorsed* suicide behaviors (SB). "X" represents endorsement of past or present SB, while "-" represents denial of ever experiencing past or present suicidal thoughts and behaviors.

Baseline	1 year follow-up	2 year follow-up	N (count)	%
x	x	x	22	8
x	-	x	19	7
x	x	-	24	9
x	-	-	121	46
-	x	-	76	29
